# Delay Selection by Spike-Timing-Dependent Plasticity in Recurrent Networks of Spiking Neurons Receiving Oscillatory Inputs

**DOI:** 10.1371/journal.pcbi.1002897

**Published:** 2013-02-07

**Authors:** Robert R. Kerr, Anthony N. Burkitt, Doreen A. Thomas, Matthieu Gilson, David B. Grayden

**Affiliations:** 1 NeuroEngineering Laboratory, Department of Electrical and Electronic Engineering, The University of Melbourne, Melbourne, Victoria, Australia; 2 Centre for Neural Engineering, The University of Melbourne, Melbourne, Victoria, Australia; 3 Bionics Institute, Melbourne, Victoria, Australia; 4 Department of Mechanical Engineering, The University of Melbourne, Melbourne, Victoria, Australia; 5 Laboratory for Neural Circuit Theory, RIKEN Brain Science Institute, Saitama, Japan; Research Center Jülich, Germany

## Abstract

Learning rules, such as spike-timing-dependent plasticity (STDP), change the structure of networks of neurons based on the firing activity. A network level understanding of these mechanisms can help infer how the brain learns patterns and processes information. Previous studies have shown that STDP selectively potentiates feed-forward connections that have specific axonal delays, and that this underlies behavioral functions such as sound localization in the auditory brainstem of the barn owl. In this study, we investigate how STDP leads to the selective potentiation of recurrent connections with different axonal and dendritic delays during oscillatory activity. We develop analytical models of learning with additive STDP in recurrent networks driven by oscillatory inputs, and support the results using simulations with leaky integrate-and-fire neurons. Our results show selective potentiation of connections with specific axonal delays, which depended on the input frequency. In addition, we demonstrate how this can lead to a network becoming selective in the amplitude of its oscillatory response to this frequency. We extend this model of axonal delay selection within a single recurrent network in two ways. First, we show the selective potentiation of connections with a range of both axonal and dendritic delays. Second, we show axonal delay selection between multiple groups receiving out-of-phase, oscillatory inputs. We discuss the application of these models to the formation and activation of neuronal ensembles or cell assemblies in the cortex, and also to missing fundamental pitch perception in the auditory brainstem.

## Author Summary

Our brain's ability to perform cognitive processes, such as object identification, problem solving, and decision making, comes from the specific connections between neurons. The neurons carry information as spikes that are transmitted to other neurons via connections with different strengths and propagation delays. Experimentally observed learning rules can modify the strengths of connections between neurons based on the timing of their spikes. The learning that occurs in neuronal networks due to these rules is thought to be vital to creating the structures necessary for different cognitive processes as well as for memory. The spiking rate of populations of neurons has been observed to oscillate at particular frequencies in various brain regions, and there is evidence that these oscillations play a role in cognition. Here, we use analytical and numerical methods to investigate the changes to the network structure caused by a specific learning rule during oscillatory neural activity. We find the conditions under which connections with propagation delays that resonate with the oscillations are strengthened relative to the other connections. We demonstrate that networks learn to oscillate more strongly to oscillations at the frequency they were presented with during learning. We discuss the possible application of these results to specific areas of the brain.

## Introduction

Spike-timing-dependent plasticity (STDP) is an experimentally observed learning rule that changes synaptic strengths based on the relative timing of pre- and post-synaptic spikes (action potentials) [1]–[4]. Gerstner et. al. first proposed it as an unsupervised Hebbian learning rule that could select feed-forward connections with specific axonal delays [5]. They showed that it could be used to achieve the high degree of temporal coherence that had been observed at frequencies of up to 8 kHz in the auditory brainstem of barn owls. This finding explained how a network could learn to perform sound localization using the time lag between the neural signals from the two ears. Their study also demonstrated that the precise timing of spikes could be captured by STDP and that this was sufficient to explain how neurons in the auditory pathway could learn to distinguish such fine temporal differences in an unsupervised fashion. In general, STDP has the ability to encode temporal correlations in neuronal activity, such as oscillations, into the functional structure of networks of neurons that have axonal and dendritic propagation delays.

The brain processes information through neuronal networks that contain specifically structured feed-forward and recurrent (lateral) connections. For example, only 5% of the input connections into cortical neurons are from the thalamus and, while these feed-forward connections tend to be strong, most of the remaining 95% are recurrent cortical connections [6], [7]. For this reason, studies of neural learning that considered recurrent networks, rather than solely feed-forward networks, offered the possibility of providing new insight into how the brain processes and encodes information. While significant work has been carried out with learning in feed-forward networks [8], [9], it was only more recently that the same attention was paid to recurrent networks [10]–[19].

Few analytical studies of spike-based learning in recurrent networks have been done, despite the ability for these studies to provide a more informative description of the mechanisms than studies that use simulations alone. A recent paper reviewed many of these studies [20]. In one such analytical study, Gilson et. al. looked at the emergent structure that forms in recurrent networks due to STDP [17]. They showed that spike correlations within two pools of inputs led to a form of symmetry breaking in the recurrent network receiving the inputs. Specifically, two sub-networks emerged with strong connections within the sub-networks but weak connections between them. In this way, the recurrent network encoded a spatial pattern of its inputs into its structure. The recurrent networks they considered contained only a narrow range of spike propagation delays. The inputs they considered contained instantaneous spike time correlations and had firing rates that were constant in time.

Most inputs and activity in the brain are, however, not constant in time. Oscillations have been observed in many different regions of the brain, such as the cortex [21]–[23] and the auditory brainstem [24]. In particular, gamma oscillations in the cortex have received considerable attention [25], [26] and have been shown to play a role in attention, memory, and other cognitive functions [21], [27]. For these reasons, it is important to consider the synaptic changes that occur due to these oscillations. Doing so may help elucidate the possible functions that oscillations play in cognitive processes.

A number of studies have explored the interaction between oscillatory activity and STDP using numerical simulations [28], [29], but only few have performed analytical investigations. Pfister and Tass considered how STDP in recurrent networks can produce stable states of high and low synchrony (oscillations) [30]. They also examined how external stimulation can force the network out of a highly synchronous state into a state of lower synchrony. Muller et. al. investigated how STDP can modify excitatory feed-forward connections into a single post-synaptic neuron such that it becomes phase-locked to oscillations in the inputs [31]. Gilson et. al. demonstrated a similar result for excitatory and inhibitory feed-forward connections with a range of dendritic delays [32]. They further showed that the post-synaptic neuron became selective in its response to oscillatory inputs at the training frequency. These studies, however, did not consider networks that have a wide range of delays on the same timescale as the oscillation period, where the correlations due to the oscillations could drive delay selection. Gerstner et. al. considered this situation for a specific neural system [5], but only for feed-forward connections and very high frequency oscillations. Though not specifically for oscillatory activity, further analysis has been performed for this concept of delay selection through STDP, although still only for feed-forward connections [33].

The broad question that motivated this study was: what can be inferred about the ways that the brain learns patterns and processes information, given the role that STDP plays in determining network structure? We specifically aim to address this for networks that have oscillatory firing patterns and a wide range of propagation delays, both axonal and dendritic. We investigate how additive STDP changes the strength of recurrent connections with a wide range of axonal delays and short dendritic delays when the network is driven by input spike trains that have oscillatory firing rates. We then look at how these changes affect the oscillatory firing rate response of the network to inputs with different oscillation frequencies. We consider a range of oscillation frequencies from 100 to 300 Hz. We discuss how this delay selection mechanism may suggest a possible explanation for how the auditory brainstem performs missing fundamental pitch perception.

We extend this simple situation and compared it to a network with a range of dendritic as well as axonal delays. We also extend the original model to one with multiple groups of neurons that are recurrently connected with connections that have a range of axonal delays. In this case, the oscillatory inputs to each of the groups have the same frequency but are out of phase with each other. In both of these cases, we focus on frequencies in the gamma range (30–100 Hz) [27]. We discuss how the second of these cases is relevant to the formation of oscillatory neuronal ensembles.

Throughout this study, we determine or estimate both the learning and dynamics of the networks analytically using the Poisson neuron model. We use numerical simulations with networks of leaky integrate-and-fire (LIF) neurons to support the results and conclusions. In the analysis and simulations, we consider only excitatory networks (i.e., without inhibition) to facilitate the mathematical analysis. We address the implications of this for the model in different contexts.

## Methods

### Poisson Neuron Model

Our analytical work used the Poisson neuron model [8]. This is a stochastic model which outputs a spike train that is a realization of an inhomogeneous Poisson process. The intensity function of this process is analogous to the membrane potential of the neuron. It is made up of a spontaneous rate and the weighted sum of post-synaptic response kernels given by
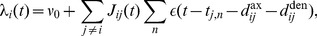
(1)where 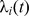 is the intensity function for the 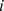
th neuron at time 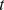, 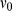 is the spontaneous rate (assumed to be zero in this study), 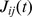 is the synaptic weight from neuron 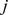 to neuron 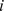, 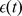 is the post-synaptic response kernel, or excitatory post-synaptic potential (EPSP) kernel, 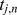 is the time of the 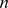
th spike output by neuron 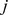, and 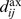 and 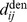 are the axonal and dendritic delays, respectively, from neuron 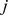 to neuron 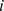. Synapses here are modeled as current based. This means that synaptic input into the neuron is independent of the neuron's membrane potential (the intensity function in this model).

In this paper, input spike trains are denoted 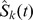, neuron spike trains are 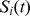, and both of these are represented as the sum of Dirac delta functions positioned at the times of spikes. These spike trains are realizations of the intensity functions, 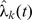 and 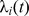, respectively, and have temporally averaged firing rates (or mean firing rates), 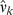 and 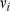, respectively.

All EPSP kernels used in this study are of the form given by
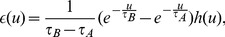
(2)where 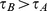 and 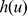 is the Heaviside function such that for 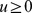, 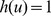, and 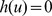 otherwise. There are three main EPSP kernels used in this study: ‘slow’, ‘medium’, and ‘fast’. The values of the time constants for these EPSP kernels are shown in Table 1.

**Table 1:** Model parameters.

Type	Parameter	Slow	Medium	Fast
EPSP	Synaptic Rise Time, 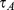 (ms)	1	0.5	0.1
EPSP	Synaptic Decay Time, 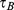 (ms)	5	1	0.5
LIF	Membrane Time Constant, 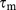 (ms)	20	10	5
LIF	Threshold Potential, 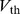 (mV)	−50		
LIF	Resting Potential, 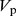 (mV)	−65		
LIF	Reset Potential, 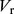 (mV)	−65		
LIF	Synaptic Reversal Potentials, 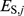 (mV)	0		
LIF	Refractory Period (ms)	1		
STDP	Potentiation Factor, 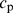	15		
STDP	Depression Factor, 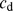	10		
STDP	Potentiation Time Constant, 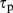 (ms)	17		
STDP	Depression Time Constant, 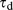 (ms)	34		

Parameters used in the model for the three different EPSPs (‘slow’, ‘medium’ and ‘fast’). All parameters were used in simulations, but only EPSP and STDP parameters were used in the analytical model.

### Spike-Timing-Dependent Plasticity (STDP)

**Figure 1:**
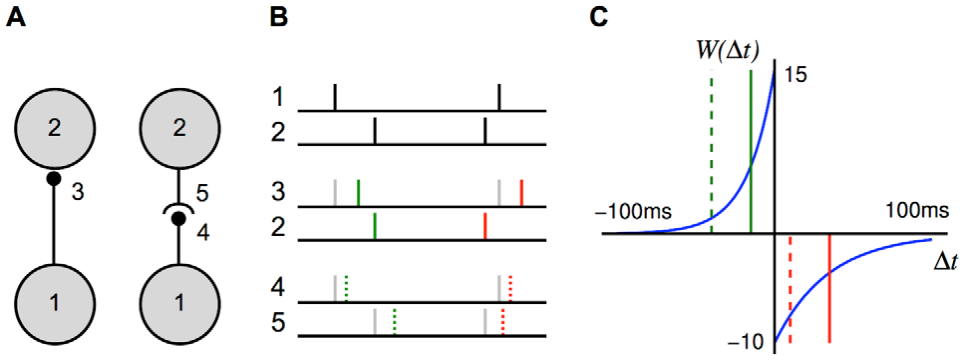
Additive STDP learning window. (A) Examples of two different synapses between the pre-synaptic neuron, 1, and the post-synaptic neuron, 2. The left one has a short dendritic delay and the right one has similar axonal and dendritic delays. (B) Examples of spike pairs. Top: Spike times are given at the somas of each of the neurons in each case in B. Middle: Pre- and post-synaptic spike times for the synapse with a short dendritic delay in B. Bottom: Pre- and post-synaptic spike times for the synapse with similar axonal and dendritic delays in B. (C) The learning window, 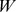, used in this study that describes how the change in synaptic weight depends upon the difference in time between the pre- and post-synaptic spikes at the synapse. The form of this is described in Equation (4) with parameter values shown in Table 1. This window was used in an additive STDP learning rule along with two rate-based terms as described in Equation (3). The changes in synaptic strength due to the synaptic spike pairs (shown in B) for each of these two cases is shown by the red and green vertical lines. This shows that as the dendritic delay is increased, or the axonal delay decreased, the 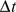 for the spike pairs is shifted to the left on the learning window (the opposite occurs for increasing the axonal delay, or decreasing the dendritic delay).

In this study, learning refers to changes made to the network due to the additive STDP learning rule [17]. The change in synaptic weight, 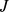, due to this rule is
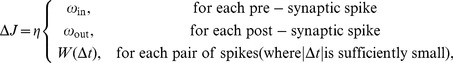
(3)where 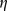 is the learning rate, 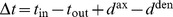, 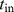 and 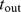 are the times of the spikes at the somas of the pre- and post-synaptic neurons, respectively, and 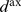 and 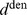 are the axonal and dendritic delays of the synapse, respectively. This is illustrated in Figure 1A and B. Finally, 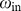 and 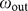 are rate-based parameters that change the synaptic weight for every pre- and post-synaptic spike, respectively. The learning window, 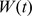, is of the form
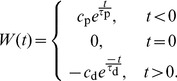
(4)where the values of the parameters used in this study are shown in Table 1. Figure 1C shows this learning window.

For a network with only axonal delays (i.e., the dendritic delays are sufficiently short to be neglected), the learning rule described in Equation (3) can be reformulated to give the rate of change of the weight 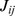 as
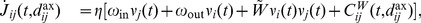
(5)where 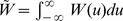, and
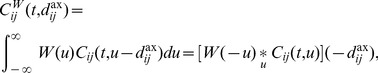
(6)where 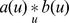 denotes the convolution of functions 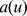 and 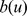 with respect to 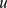. The axonal delay, 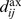, can be seen to effectively shift the learning window in a positive direction. The correlation function for a pair of neurons in a recurrent network is defined by [20]

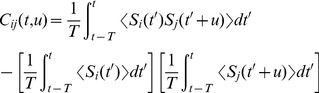
(7)This notation generalizes that used previously [17], in which only constant input intensity functions were considered.

### Network Configuration

**Figure 2:**
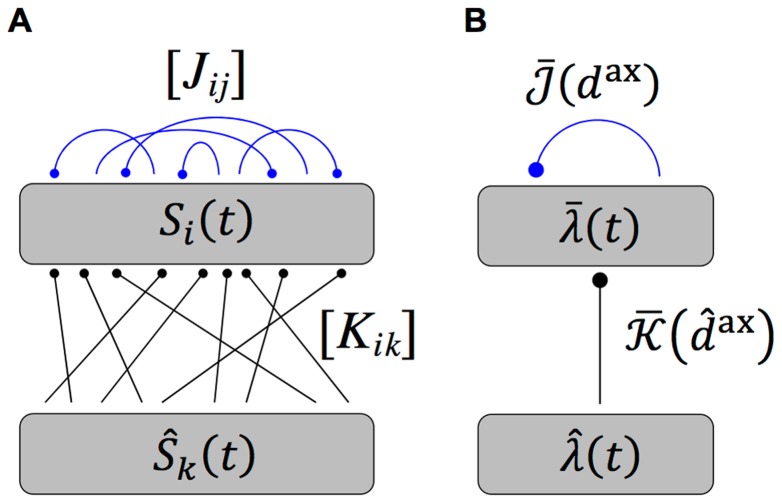
Diagram of single group network model. (A) Diagram of the full model used in simulations, which shows a network of 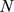 neurons with spike trains, 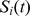, that receive inputs from 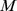 inputs, 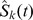, via fixed (black), feedforward connections denoted by 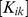, and from each other via plastic (blue), recurrent connections denoted by 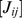. (B) Diagram of the simplified, analytical model, which shows the same network represented by an ensemble averaged, instantaneous firing rate, 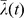, which is driven by inputs with instantaneous firing rate, 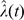. The (fixed; black) feedforward and (plastic; blue) recurrent connections are represented by the axonal delay profiles, 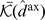 and 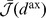, respectively.

The network configuration that we considered, as illustrated in Figure 2A, consisted of a single network of 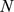 neurons. Each neuron received feed-forward connections from a set of 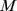 inputs and also recurrent connections from other neurons in the network. The inputs were spike trains, 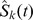, each a different realization of the same Poisson process with intensity function, 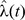. This intensity function was oscillatory in time; it can be thought of as the instantaneous firing rate of the inputs with mean (temporally averaged) firing rate, 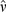. Each neuron received 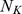 feed-forward connections, all with the same weight, 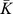, and axonal delay, 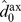, and negligible dendritic delays. The neurons each produced spike trains, 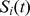, according to the neuron model used. In this study, this was either the Poisson neuron model or the leaky integrate-and-fire (LIF) neuron model. There were 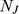 recurrent connections into each neuron. These were initially all the same weight but were modified by additive STDP. These connections each had different axonal delays, sampled uniformly from a range. Initially, we assumed these connections had negligible dendritic delays. This model is illustrated in Figure 2A, where 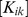 and 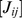 denote the matrices of feed-forward and recurrent connections just described.

In this study, we always used (unless otherwise stated) 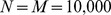, 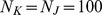, and 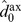 to 1 ms. The axonal delay range (and later the dendritic delay range) used in this study was 1–10 ms. This is consistent with the magnitude of axonal delays observed in the cortex (4–20 ms) [34], while perhaps less so for the auditory brainstem (0.4–1.4 ms) [35].

When the neurons were modeled using the Poisson neuron model, we simplified the full model analytically in two major ways. This simplification is illustrated in Figure 2B. First, instead of the full set of input and neuron spike trains, we considered only the ensemble averaged, instantaneous firing rates, 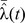 and 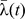, for the input and network neurons, respectively (as the inputs have identical intensity functions, 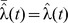). Second, we represented the sets of feed-forward and recurrent connections as weighted axonal delay profiles (or simply axonal delay profiles or delay profiles), 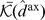 and 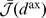, respectively. These delay profiles give the mean weight for connections with a specific axonal delay (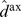 or 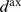, respectively). When representing a set of recurrent connections that are uniformly sampled from a fixed range of axonal delays (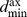 to 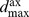), the integral of the recurrent axonal delay profile is
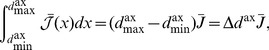
(8) where 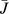 is the mean recurrent weight in the network and 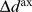 is the range of the axonal delays in the network. We relaxed our definition of the axonal delay profile representing the mean weight for a specific axonal delay when the range of the axonal delays, 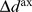, was zero. This is the case for the input connections, as they all have the same axonal delay, 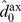. The profile is instead given by 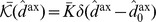, where 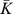 is the mean feed-forward weight (and also the integral of the profile). Other feed-forward delay profiles (e.g. Gaussian) could have been considered but this was the simplest analytically and the effect of other profiles would solely be to reduce the effective modulation amplitude of the input spike trains.

This study investigated the learning that occurs in the recurrent network through changes in the recurrent axonal delay profile. It also considered the amplitude of the oscillatory firing rate of the network (averaged over the neurons in the network) to different oscillatory inputs after this learning has occurred.

### Learning with Axonal Delays

We investigated the learning of the recurrent connections in the network by considering the changes to the recurrent axonal delay profile due to Equation (5). We modified Equation (5) to apply to the recurrent axonal delay profile. The new learning equation is
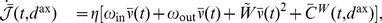
(9)where 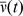 is the temporally averaged firing rate of the recurrent neurons and 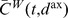 is the convolution of the learning window, 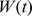, with the mean recurrent correlation function, 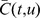. The first three terms in this equation determine the evolution of the mean recurrent weight over all axonal delays. We were interested in the last term, which determines the average deviation from this mean for connections with different axonal delays. In this model, learning was assumed to happen on a longer timescale compared with that of the network activity and so we treated the recurrent correlation as quasi-stationary. For this reason, the 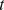-dependence of the average recurrent correlation function is dropped and so is given by 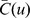 in the subsequent analysis of this paper. Using the simplified model with a recurrent axonal delay profile, we found the (ordinary frequency) Fourier transform of 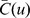 (see Section 1 of Supporting Text 10) to be approximated by
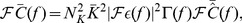
(10)where
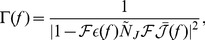
(11)

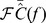
 is the Fourier transform of the average input correlation function, 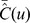, 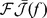 is the Fourier transform of the axonal delay profile, 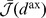, 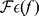 is the Fourier transform of the EPSP kernel, 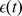, and 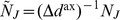.

The input intensity function for a population of oscillatory inputs is defined as
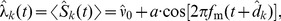
(12)where 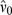 is the mean input rate (in spikes/s), 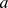 is the amplitude in the oscillations (in spikes/s), 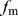 is the modulation frequency of the oscillations (in Hz), and 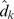 is the delay of the input (in seconds). In this model, all inputs are assumed to be in phase with each other. As Section 2 of Supporting Text 10 shows, the temporally averaged input firing rate is 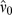 and the correlation function for any pair of inputs is
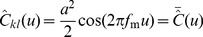
(13)and the Fourier transform of this is
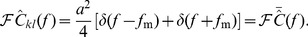
(14)It should be noted that no additional higher-order spike timing correlations were introduced in the input spike trains. The correlations described here are the rate correlations arising solely from the fact that all input neurons shared a common firing rate modulation.

With oscillatory inputs, the average recurrent correlation function becomes
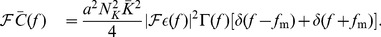
(15)


It can be seen that 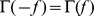 and 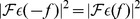. Using this, the Fourier transform of the correlation function can be combined with the learning window (shifted by the axonal delay), as described by Equation (6), to give the contribution to learning from recurrent correlations for connections of axonal delay, 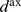, as
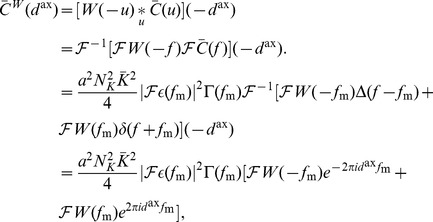
(16)where 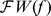 is the Fourier transform of the learning window, 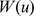.

This was reformulated, by rewriting 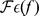 as 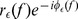 and 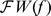 as 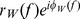, to be
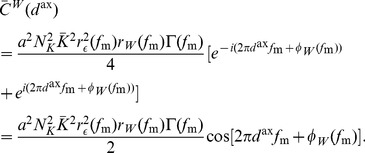
(17)Expressions for functions 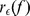, 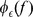, 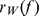, and 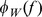 were derived, for the specific EPSPs and learning window used in this study, in Section 6 of Supporting Text 10.

Assuming weak recurrent connections compared to the input connections, 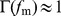, we derived the approximation
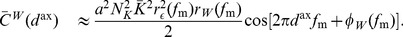
(18)


The deviation of the mean weight for a given delay, 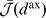 from the mean weight over all delays, 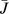, is defined as 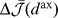. Although, the mean weight is driven towards a homeostatic equilibrium, 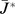, by the rate-based learning terms (see Section 3 of Supporting Text 10), the evolution of the deviation of weights from this mean is described by
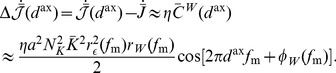
(19)


### Response to Oscillations after Axonal Delay Selection

To determine the response of a network, after learning, to oscillatory inputs, we first needed to consider the instantaneous firing rate of a single neuron, which is given by
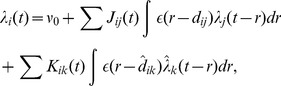
(20)where 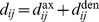 and 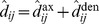 are the total recurrent and input delays, respectively, which are the sums of the axonal and dendritic delay components. This means that the network response only depends on the total delays, not directly on the axonal or dendritic components, so only total delays are referred to in the following derivation. In networks with short dendritic delays, the axonal delay is equivalent to the total delay.

We assumed that the input connections have equal total delay, 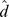, the inputs have identical rate functions, 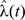, and 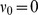. We also represented all the recurrent weights as a profile over total delay, 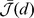. Therefore, the average response of the network is
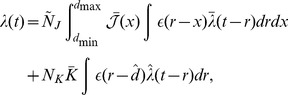
(21)where 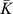 is the mean feedforward weight.

For oscillatory inputs, 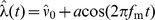, we showed that the expression for the response of the network becomes (see Section 4 of Supporting Text S1)
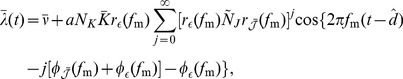
(22)where 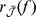 and 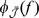 are defined by
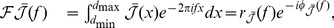
(23)and
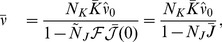
(24)and, as before, 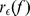 and 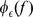 are given by Fourier transform, 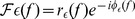.

To the second order, we approximated the network response as
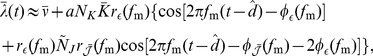
(25)and, since 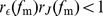, we approximated the network response amplitude as
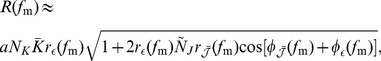
(26)using the result from Section 7 of Supporting Text S1.

We assumed a Gaussian delay profile with mean, 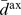, standard deviation, 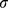,
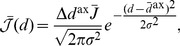
(27)so we found that
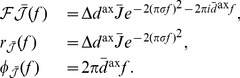
(28)We found the amplitude of the response function with this Gaussian delay profile by substituting Equation (28) into Equation (26).

### Learning with Both Axonal and Dendritic Delays

As previously considered [18], when dendritic delays are included together with the axonal delays, the expression for the learning term, 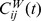, becomes
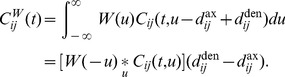
(29)


We performed a similar derivation as for learning with only axonal delays. We found that, for oscillatory inputs, the learning term due to correlations is a function of both the axonal and dendritic delays,
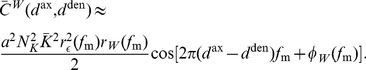
(30)


Therefore, the deviation of the mean weight for a given axonal and dendritic delay evolves according to
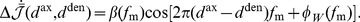
(31)


### Learning with Two Recurrently Connected Groups

**Figure 3:**
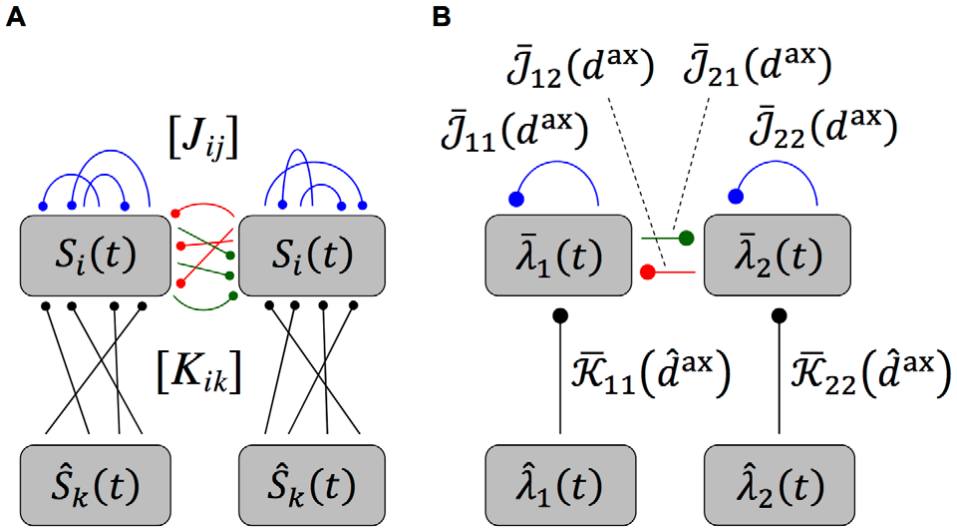
Diagram of two group network model. (A) Diagram of the full model used in simulations, which shows a network of 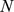 neurons with spike trains, 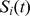, divided into two groups that each receive inputs from a different group of 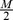 inputs, 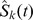, via fixed (black), feedforward connections, denoted by 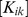, and from each other via plastic (blue, red and green), recurrent connections, denoted by 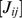. (B) Diagram of the simplified, analytical model, which shows the same network represented by an ensemble averaged, instantaneous firing rate for each group, 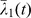 and 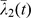, respectively, that are driven by inputs with instantaneous firing rates, 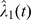 and 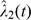, respectively. The (fixed; black) feedforward and (plastic; blue, red, and green) recurrent connections are represented by the axonal delay profiles, 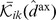 and 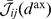, respectively, where 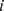 denotes the group that the connections are to and 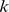 or 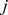 denote the group of inputs or neurons that the connections are from.

The recurrent network was also considered to be made up of two groups of neurons with each group receiving inputs from a different group of oscillatory inputs, as shown in Figure 3. We once again considered networks with short dendritic delays.

Here, 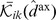 and 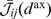 are defined as the mean feed-forward and recurrent weights from group 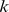 or 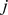 to group 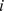 with delay 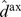 or 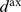, respectively. We considered the case of two network groups, each with 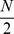 neurons, and two input groups, each with 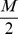 spike trains. The input connection matrix, 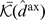, is defined for the two input groups and two recurrent groups as
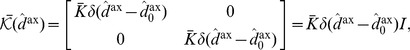
(32)where, as before, 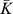 and 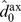 are the mean feed-forward weight and the axonal delay of input connections, respectively. The spike trains in each input group were generated from the group's input intensity function. These are defined for each group of oscillatory inputs as
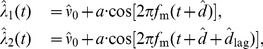
(33)where 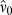 is the mean input rate (in spikes/s), 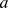 is the amplitude in the oscillations (in spikes/s), 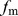 is the modulation frequency of the oscillations (in Hz), 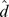 is the delay of inputs in the first group (in seconds), and 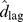 is the time lag between the oscillations of the two input groups (in seconds). We determined that the average input correlation function matrix is (see Section 2 of Supporting Text S1)
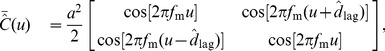
(34)and the Fourier transform is
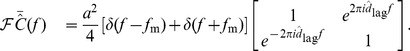
(35)


As with learning for a single group, we assumed weak recurrent connections. Therefore, we approximated the Fourier transform of the average recurrent correlation function as
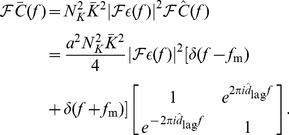
(36)


Therefore,
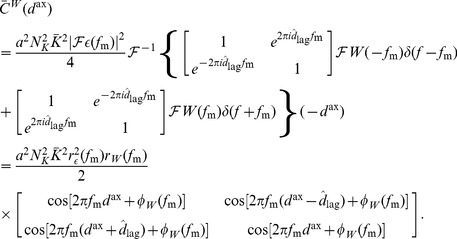
(37)


### Response of Two Groups after Axonal Delay Selection

For two recurrently connected groups, where the within group weights have been depressed and the inputs are as in Equation (33), each of the group responses is
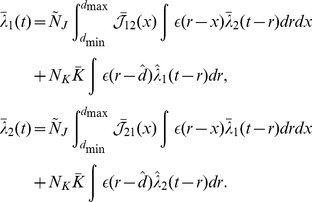
(38)


As derived in Section 5 of Supporting Text S1, we approximated this as
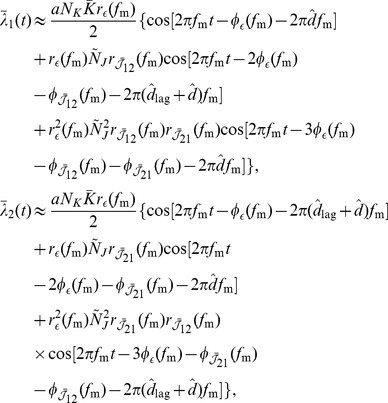
(39) where 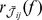 and 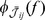 are the amplitude and negative phase of the Fourier transform of the axonal delay profile of connections from group 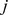 to group 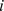, respectively. As we did for a single recurrent group, we assumed the between group delay profiles were Gaussian. Specifically, it was assumed that 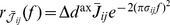 and 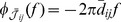. The result from Section 7 of Supporting Text S1 was used to approximate the amplitude of this response.

### Numerical Simulations

Simulations were performed using the leaky integrate-and-fire (LIF) neuron model. A single state variable, 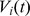, represents the membrane potential for each neuron 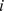 that evolves according to
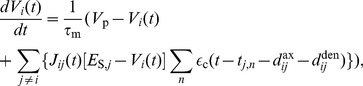
(40)where 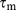 is the passive membrane time constant, 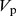 is the resting membrane potential, 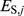 is the synaptic reversal potential of the (excitatory) synapses from neuron 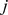, and 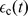 represents the excitatory post-synaptic conductance (EPSC). This plays a similar role to the EPSP kernel, 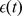, in the Poisson neuron model and, because of this, we refer to both 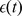 and 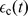 as the EPSP or the EPSP kernel. 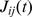, 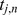, 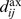 and 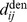 are the same as for the Poisson neuron model. A spike was produced when the membrane potential reached a threshold value, 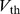, and it was reset to 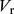. An absolute refractory period was used, which prevented the membrane potential from changing during this time. The values of these parameters are shown in Table 1. It should be noted that different values for the membrane time constant were used for the three different EPSP kernels considered. Simulations were of a model with 10,000 LIF neurons that each received 100 randomly chosen input spike trains from a total of 10,000. These neurons also received 100 recurrent connections from other neurons in the network, which had no dendritic delay and axonal delays that were sampled uniformly from the range 1–10 ms. The weights of the input (feed-forward) connections were fixed and chosen to be equal to each other and such that, without recurrent connections, the temporally averaged firing rate of the neurons was approximately equal to that of the inputs. The weights of the recurrent connections were updated by STDP during the simulation. They were initialized to be equal to each other and such that they significantly increased the firing rate of the neurons above the base rate caused by the inputs alone. Simulations were performed using an in-house neuron modeling software program, SpikeSim, used in previous studies [17], [18].

The networks simulated and considered in this paper contained only excitatory neurons and operated in super-threshold, mean-driven regimes. We address this and consider the limitations for this as a model of networks in the auditory brainstem or the cortex in the Discussion.

## Results

In this study, we considered how STDP leads to delay selection in the recurrent connections of a network receiving oscillatory inputs. We used the Poisson neuron model to derive analytical results and the leaky integrate-and-fire (LIF) neuron model in simulations. The observed learning was due to additive STDP together with single-spike contributions, or rate-based terms. As in previous work [17], these rate-based terms, along with the learning window cause the mean recurrent weight to converge to a homeostatic equilibrium. However, in this study, we were concerned with the deviation of individual weights with specific delays from the mean weight.

### Axonal Delay Selection within a Recurrent Network

We first considered how STDP changes the functional connectivity of a recurrent network receiving inputs from a single group of oscillatory inputs. The connections in the networks had a range of axonal delays (1–10 ms) but very short dendritic delays. The modulation frequencies of the inputs were between 100 and 300 Hz. This range is typical of the fundamental frequency of sounds encoded in modulation frequencies in the auditory brainstem. We modeled the recurrent connections as a weighted axonal delay profile 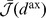, which is the mean weight of connections with a given axonal delay. We analytically derived an expression for the changes made to this profile and showed that these predicted changes were supported by numerical simulations.

As detailed previous studies [17], the rate-based plasticity parameters 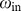 and 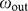, together with the learning window bias 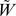, caused the mean weight in the network to converge to a stable equilibrium value, 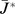. The mean weight of connections with a given axonal delay deviated from this homeostatic equilibrium as given by, 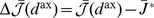. For inputs with a given modulation frequency, 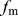, we predicted that this deviation would evolve according to (see Equation (19))
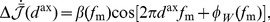
(41)where 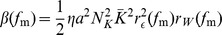 is a positive factor that determines the rate of learning, 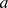 is the amplitude of the input modulation, 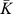 is the mean feed-forward weight. The functions 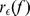 and 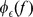 denote the amplitude and negative phase of the Fourier transform of 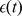 (i.e. 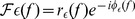), respectively. The functions 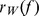 and 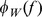 denote the amplitude and phase of the Fourier transform of 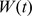 (i.e. 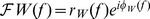), respectively. A plot of 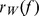 is shown in Figure S1. The functions 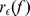 and 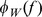 are considered in more detail in the next section.

**Figure 4:**
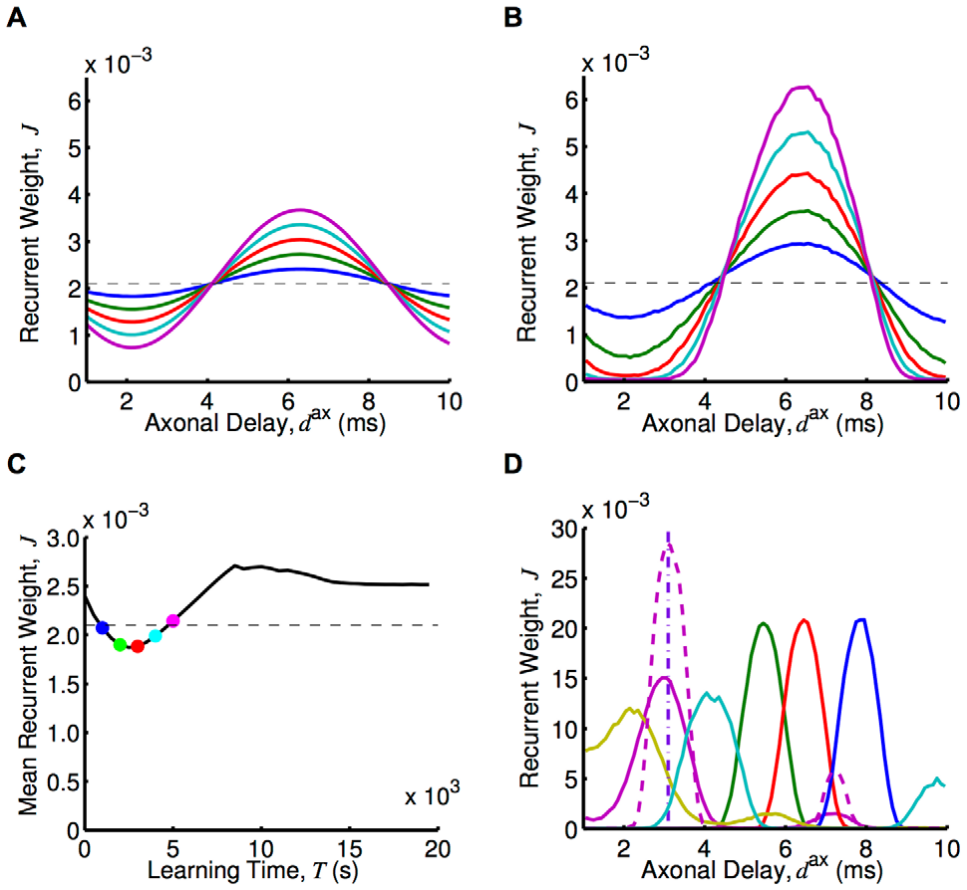
Learning through axonal delay selection with oscillatory inputs. (A) Axonal delay profiles predicted by the analytical model, Equation (41) (which uses the Poisson neuron model), with an input modulation frequency of 120 Hz, after 1000s (blue), 2000s (green), 3000s (red), 4000s (cyan), and 5000s (magenta) of learning from the initial (dashed) profile. (B) As for A, but for a simulation using LIF neurons. (C) Mean recurrent weight, for connections of any axonal delay, over time for the simulation in B (black) and the stable mean recurrent weight assumed in the analytical model (dashed), with colored dots showing the mean recurrent weight in the simulation for the times corresponding to the profiles shown in B. (D) Axonal delay profiles after 20,000s of learning in simulations with LIF neurons for input modulation frequencies of 100 Hz (blue), 120 Hz (red), 140 Hz (green), 180 Hz (cyan), 240 Hz (magenta), and 300 Hz (yellow). These simulations used a ‘medium’ EPSP (solid), except for one that used a ‘fast’ EPSP (dashed). Also shown is a delta delay profile at 3.1 ms (purple, dot-dashed). For both analytical and simulations, a modulation amplitude, 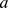, of 5 spikes/s was used.

Assuming upper and lower bounds on the synaptic weights, we determined the axonal delay profiles that resulted from this learning. An example of this is shown in Figure 4A for an input frequency of 120 Hz. Eventually, a narrow range of delays was uniquely selected because of the bounds on the weights. This narrow range was centered approximately on the delay that resonated with the input frequency. The shortest of these is given by
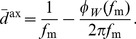
(42)If this delay is outside the range of axonal delays in the network, then the frequency cannot be encoded by the network. The minimum (maximum) delay in the recurrent connections of the network sets the limit on the maximum (minimum) frequency that can be learned.

Equation (41) shows that the synaptic rise and decay times only affect the learning rate, 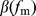, and not the delays that are selected. The learning rate is also dependent upon the square of the amplitude of oscillations and the square of the input strength. For the simulations with LIF neurons, the firing rate of neurons is no longer linear with the input strength so this learning rate dependence on input strength is different but it is still a non-decreasing dependence.

We compared the learning that occurs with 120 Hz inputs, shown analytically in Figure 4A, to simulations with 10,000 LIF neurons. This is shown in Figure 4B. The shape of the delay profile learned was the same; it is only the rate of learning that differs. The simulations with the LIF neurons showed a significantly faster learning rate. Simulations with Poisson neurons, however, did not show this difference when compared to the analytical model (see Figure S2). The higher learning rate appears to be due to the differences between the Poisson and LIF neuron models. We saw that, after further learning occurred, the mean recurrent weight did not remain at the homeostatic equilibrium. Instead the mean recurrent weight increased up to a critical point (Figure 4C). The concept of a “critical point” and how it is relevant to the response of the network is explained in more detail in the Discussion section. For learning within the simulation, it is sufficient to observe that the mean recurrent weight increased above the homeostatic equilibrium, providing another way that the simulation differed from the analytical model.

We observed that different delays were selected for different input frequencies. This is shown in the delay profiles in Figure 4D. These are the result of 20,000s of learning in simulations with 10,000 LIF neurons. It can be seen that for the higher frequencies used (180, 240 and 300 Hz) there was a second smaller peak at a longer delay. Equation (41) predicts that this second delay (and others that are within the allowed range for axonal delays) should be equally selected for. However, the simulations showed that, while each of these delays was initially potentiated, eventually the shortest of these delays was selected over the longer ones. We used a ‘medium’ EPSP kernel (0.5 ms rise time, 1 ms decay time) in all previously mentioned simulations. The learning for 240 Hz with a ‘fast’ EPSP kernel (0.1 ms rise time, 0.5 ms decay time) is shown in Figure 4D.

### Frequency Selective Response after Delay Selection

Next, we considered how this learning changed the way the network responds to different input frequencies. Being driven by oscillatory inputs, the network always had an oscillatory response at the same modulation frequency. We derived an approximation to the amplitude of this oscillatory response, 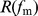, as a function of the modulation frequency of the inputs (see Equation (26))
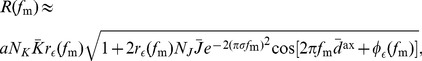
(43)where the network has a Gaussian axonal delay profile centered about 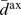 with a standard deviation of 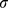 (and short dendritic delays, 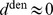). Additionally, 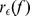 and 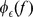 denote the amplitude and negative phase, respectively, of the Fourier transform of the EPSP kernel, 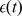 (i.e. 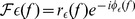) and 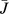 is the mean recurrent weight.

**Figure 5:**
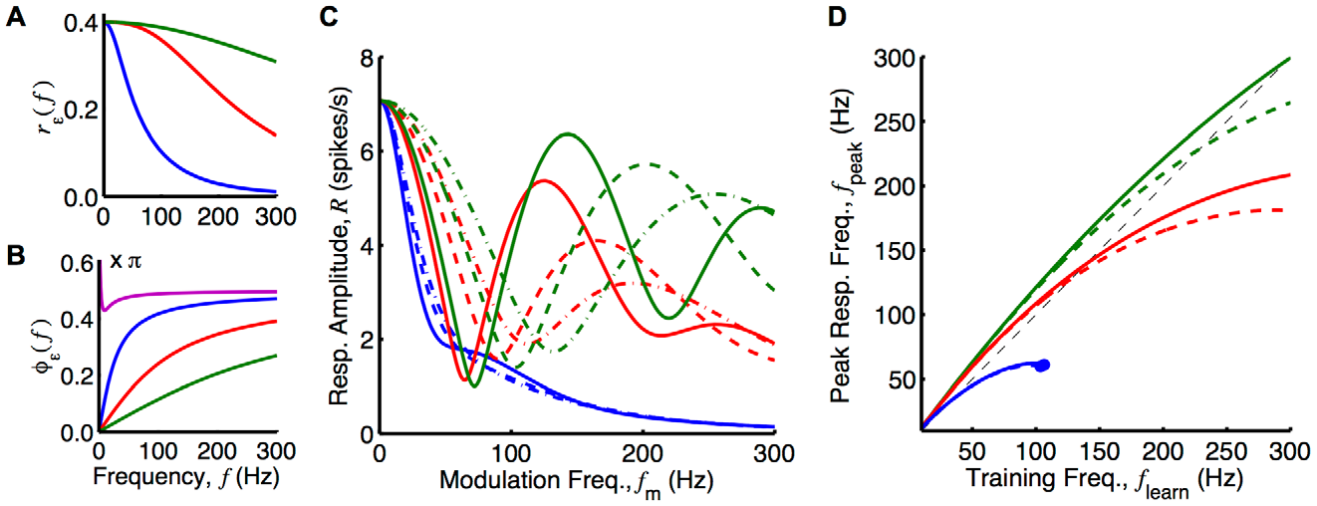
Analytical investigation of network response after delay selection. Responses considered for ‘slow’ (blue), ‘medium’ (red), and ‘fast’ (green) EPSPs. (A) Amplitude of the Fourier transform of the three EPSPs as functions of frequency, as given by Equation (33) in Section 6 of Supporting Text S1. (B) Negative phase of the Fourier transform of the three EPSPs compared to the phase of the Fourier transform of the learning window (magenta) as functions of frequency, as given by Equation (31) and (33) in Section 6 of Supporting Text S1. (C) Analytically determined response of networks with delay profiles centered about the delay selected due to learning (Equation (42)) with input frequencies of 120 Hz (solid), 180 Hz (dashed), and 240 Hz (dot-dashed) inputs, with a profile width, 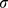, of 0.5 ms. These curves are given by Equation (43). (D) Peak response frequency, 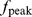, as a function of the training frequency, 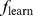, of the network, for delay profiles with a width, 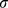, of 0.5 ms (solid) and 1 ms (dashed). The peak response frequency was numerically determined from the analytical formula in Equation (43). The dashed line represents 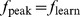. Note that the dot ending some of the lines represents that, for higher training frequencies, there was no peak in the response amplitude with frequency. For plots C and D, a recurrent strength, 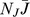, of 0.5 and a modulation amplitude, 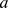, of 5 spikes/s were used.

The shape of this response function, 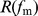, is highly dependent on the amplitude of the Fourier transform of the EPSP, 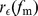, being used (see Figure 5A). This depends on the decay time and, to a lesser extent, on the rise time of the EPSP in the model (see Section 6 of Supporting Text S1). Figure 5C shows that, when the ‘slow’ EPSP (1 ms rise time, 5 ms decay time) was used, the oscillatory response of the network was very small in the frequency range considered regardless of how the delays in the network were tuned. The ‘medium’ EPSP (0.5 ms rise time, 1 ms decay time); however, gave rise to an oscillatory response with an amplitude peaked at a particular frequency. This frequency depended on the axonal delay, 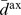, about which the profile was centered. Using the ‘fast’ EPSP (0.1 ms rise time, 0.5 ms decay time), this selective response was even more pronounced. This difference was larger when the axonal delay profile was such that the peak response amplitude was at higher frequencies.

The frequency of the peak in the response amplitude function (excluding the peak at 0 Hz) is denoted by 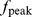. This frequency does not necessarily correspond to the frequency present during learning, 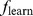. Equation (42) shows how 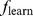 determines the selected axonal delay, 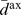. The correspondence between these two frequencies depends on the difference between 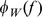 and 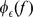. This is shown in Figure 5B for ‘slow’, ‘medium’, and ‘fast’ EPSPs. This shows that 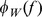 was larger than 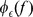 across the frequency range, for any of the EPSPs considered. This tended to cause 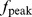 to be higher than 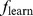. However, there is a second factor affecting the correspondence between 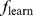 and 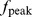. This is the decay with frequency that was evident in both 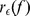 (see Figure 5A) and the 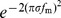 term. These decays tended to make 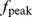 lower than 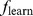. Figure 5D shows the correspondence between 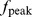 and 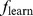, for ‘slow’, ‘medium’, and ‘fast’ EPSPs for narrow and wide delay profiles. We generated this plot by first considering the response amplitude function, 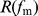, that resulted from assuming the selected axonal delay produced by the training frequency, 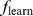. We then numerically found 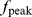 as the location of the first peak (after 0 Hz) in this function. A similar plot for different learning window parameters is shown in Figure S3. This demonstrates the robustness of the mechanism to the learning window used. It is important to note that these plots do not take into account the membrane time constant (not present in the Poisson neuron model). This was present in simulations using the LIF neuron model and worked to effectively increase 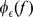, bringing it closer to 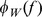. Later in this section, results of simulations with LIF neurons show how this affected the frequency of the peak response.

We compared the analytical expression for the network response amplitude for various input frequencies, 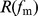, to simulations using the Poisson neuron model. We carried out simulations with networks of 10,000 neurons. These simulations were done before any learning had occurred in the network (all weights were equal) and then after 20,000s of learning with 120 Hz oscillatory inputs. The axonal delay profiles of this network before and after learning, along with a Gaussian profile fit to the after-learning profile, are shown in Figure S4A. Simulations were run multiple times, each for 10s of modeled time and with a different input modulation frequency, for frequencies ranging from 30 Hz to 300 Hz in steps of 10 Hz. The amplitude of the responses for these two networks as a function of the input modulation frequency is compared to an analytical approximation in Figure S4B. We determined this analytical approximation using Equation (43) and assuming the Gaussian delay profile that was fitted to the after-learning network. The analytical approximation closely matched the responses observed in the simulations.

**Figure 6:**
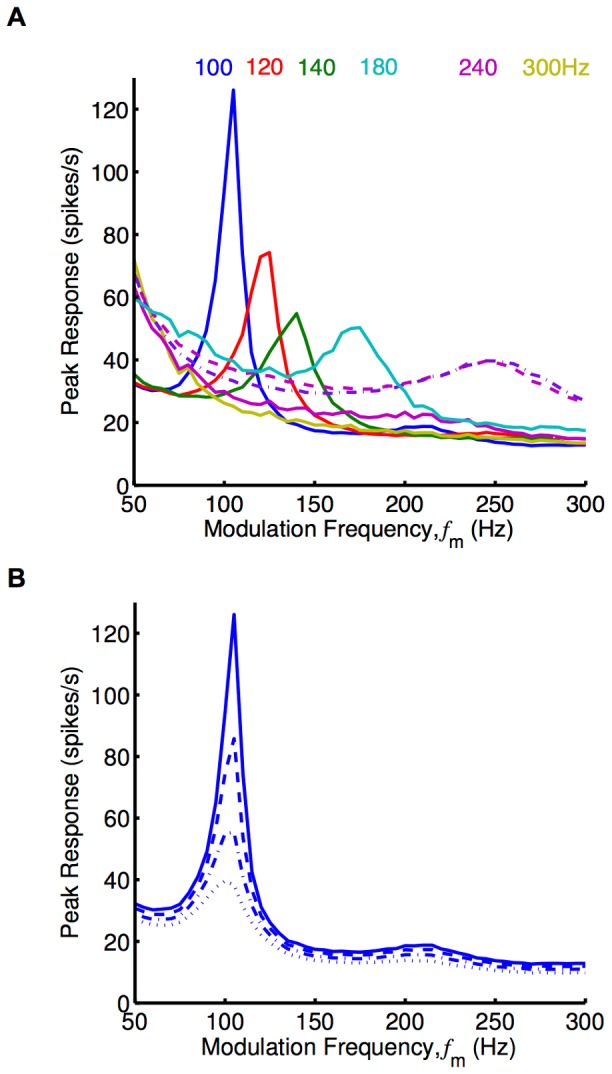
Simulations of peak network response after delay selection. Response plots showing the peak in the periodic response of networks of LIF neurons plotted as a function of the modulation frequency, 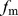 (‘medium’ EPSPs used unless otherwise specified). (A) Response plot for networks after 20,000s of learning with input modulation frequencies of 100 Hz (blue), 120 Hz (red), 140 Hz (green), 180 Hz (cyan), 240 Hz (magenta), and 300 Hz (yellow), as shown in Figure 4D. The simulations used ‘medium’ EPSPs (solid), except for two which used the fast EPSP (dashed and dot-dashed). The weights in the networks trained with 240 Hz and 300 Hz inputs were scaled down slightly (to about 0.99 of their original value) so that the networks were below criticality. (B) Response plot for the network trained with 100 Hz inputs in A, with the weights all scaled by 0.90 (dotted), 0.95 (dot-dashed), 0.98 (dashed), 1.00 (solid).

Similar to the above simulations with the Poisson neuron model, we carried out simulations using networks of 10,000 LIF neurons (and ‘medium’ EPSPs). These networks had each learnt over 20,000s while receiving inputs with different modulation frequencies (‘training frequencies’). The resulting axonal delay profiles of these networks are shown in Figure 4D. We ran simulations multiple times with all of these networks, each for 10s of modeled time and with input modulation frequencies (‘testing frequencies’) ranging from 50 Hz to 300 Hz in steps of 5 Hz. The peak instantaneous firing rates of the periodic responses (averaged over neurons), or the peak response, observed are shown in Figure 6A. The peak instantaneous firing rate is presented instead of the amplitude because the response, while still periodic with the same frequency as the input, was no longer a cosine function. This was due to the non-linear nature of the LIF model. For networks trained with 100 Hz, 120 Hz, 140 Hz, and 180 Hz, these response curves showed a clear selectivity toward the input modulation frequency at which they were trained.

While LIF networks were able to encode higher training frequencies (240 Hz, and 300 Hz) in their selected axonal delays (Figure 4D), they did not respond selectively to this frequency after learning. This was largely due to the fact that the network was not able to respond with these higher frequencies regardless of the delays in the network. We hypothesized that networks with faster synapses and neurons would be able to show a stronger response at these higher frequencies. We considered this situation by running simulations using a network with faster synapses and neurons that was trained with an input frequency of 240 Hz. This is described in the Methods section and the learned network is shown in Figure 4D. Its response is shown in Figure 6A. We observed that the network showed selectivity to an input frequency of 250 Hz. This was very close to the trained frequency. The response of the network with all of the axonal delays set to 3.1 ms (also shown in Figure 4D) showed a response with only slightly improved selectivity. Another point to notice is that the response of the networks trained with higher frequencies (180, 240, 300 Hz) to frequencies in the lower range (50–100 Hz) was higher than networks trained with 100, 120 or 140 Hz. This was likely due to the fact that the potentiated delays in these networks were relatively short. It may be that these short delays were providing recurrent feedback within the same oscillation peak, which for lower frequencies like 50 Hz was relatively wide.

The recurrent connections in a network of excitatory neurons provided positive feedback to the network. For weak recurrent connections, this positive feedback did not greatly affect the firing of the neurons in the network. As this feedback increased, these connections caused higher firing rates. This continued up to a critical point where the feedback caused the firing in the network to continue increasing in an unstable manner. A network with its mean recurrent weight at this point can be said to be critical, or to have reached criticality. The trained networks we considered ended up just below criticality after learning. Figure 6B shows the change to the response of the network caused by scaling down the recurrent weights in the network trained with 100 Hz. This shows a decreasing frequency selectively as the network moves away from criticality. In Figure 6A, it was necessary for us to scale down all of the recurrent weights in the networks trained with 240 and 300 Hz by a slight amount (down to 0.99 of their original value) so that they were below criticality (for all frequencies).

### Axonal and Dendritic Delay Selection

We extended the learning of oscillatory inputs by axonal delay selection to consider the networks with connections that had a range of dendritic as well as axonal delays. To do this, we needed to consider the recurrent connections as a weighted delay profile over both axonal and dendritic delays, 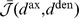. We derived an expression for how this evolves due to STDP (see the Methods section). As previously, we predicted the evolution of the deviation of this profile from the homeostatic equilibrium
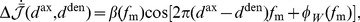
(44)where, as before, 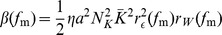.

**Figure 7:**
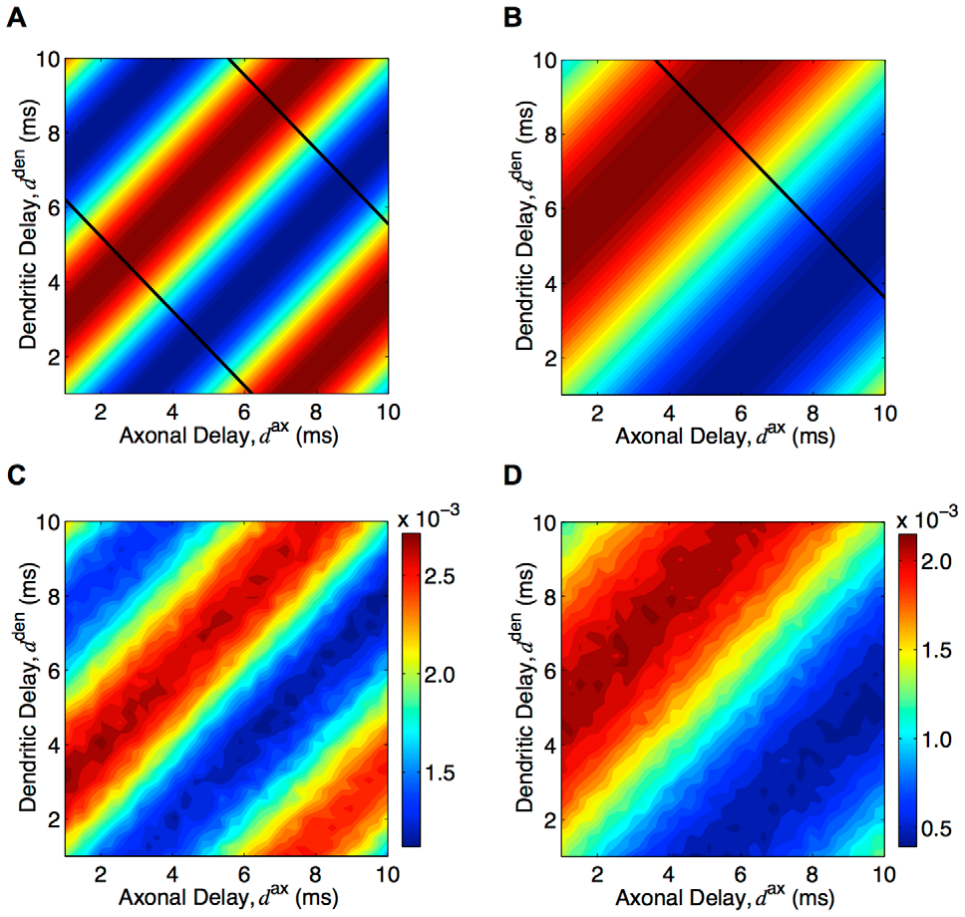
Axonal and dendritic delay selection. (A) Analytically determined heat map of potentiation/depression of connections with different axonal and dendritic delay with 120 Hz inputs and ‘medium’ EPSP, as given by Equation (44). Regions of red correspond to potentiation and regions of blue correspond to depression. Black lines correspond to the delays that maximize the response at 120 Hz. (B): Same as A but with 60 Hz inputs and ‘slow’ EPSPs (black lines correspond to the delays that maximize the response at 60 Hz). (C) Resulting heat map of mean connection strengths for different axonal and dendritic delays after simulating 500s of learning with 10,000 LIF neurons, ‘medium’ EPSPs, and 120 Hz inputs. (D) Same as C but with ‘slow’ EPSPs, 60 Hz inputs, and learning for only 50s. Note that no color bars are shown in A and B as the value of the weights is arbitrary; the mean depended on the homeostatic equilibrium and the amplitude on the learning duration.

This analytic result can be visualized using a heat map of the deviation of weights from the homeostatic equilibrium for connections of different axonal and dendritic delays. Figure 7A shows the resulting heat map after learning with ‘medium’ EPSPs and inputs with modulation frequencies of 120 Hz. This same result is shown in Figure 7B for gamma frequency inputs (60 Hz) and ‘slow’ EPSPs (typical of pyramidal neurons) to model how this mechanism may work in the cortex. In both of these cases, the two-dimensional delay profiles that were learned showed a bias towards connections that have a linear relationship between their axonal and dendritic delays (with a slope of 1.0). We compared these analytic results (Figure 7A and B) to simulations of networks of 10,000 LIF neurons. As shown in Figure 7C and D, these results supported the analytic model.

In order for the network to show a selective response, it is the sum of the axonal and dendritic delays (not the difference between them) that is required to be tuned to a particular value. The diagonal black lines in Figure 7A and B show the connections that have the specific axonal and dendritic delays required for the network to have its largest response amplitude at the training frequency. It can be seen that these lines did not match at all with the delays selected during learning. The implications of this are addressed in the Discussion section.

### Delay Selection with Multiple, Out-of-phase, Oscillatory Groups

Gamma oscillations (40–60 Hz) are the highest modulation frequencies typically observed in the cortex. Axons within a single group of neurons would need to have delays of approximately 10–20 ms to be selected by the STDP delay selection mechanism considered thus far. This may not be realistic for most axons in the cortex. We showed that for multiple groups of neurons receiving out-of-phase, oscillatory inputs it was possible for multiple, shorter delays (e.g. 1–10 ms) to encode these lower frequencies (e.g. in the gamma range). More interestingly, these delays could simultaneously encode the time lag between the groups. These different groups of neurons can be thought of as being in different cortical regions.

**Figure 8:**
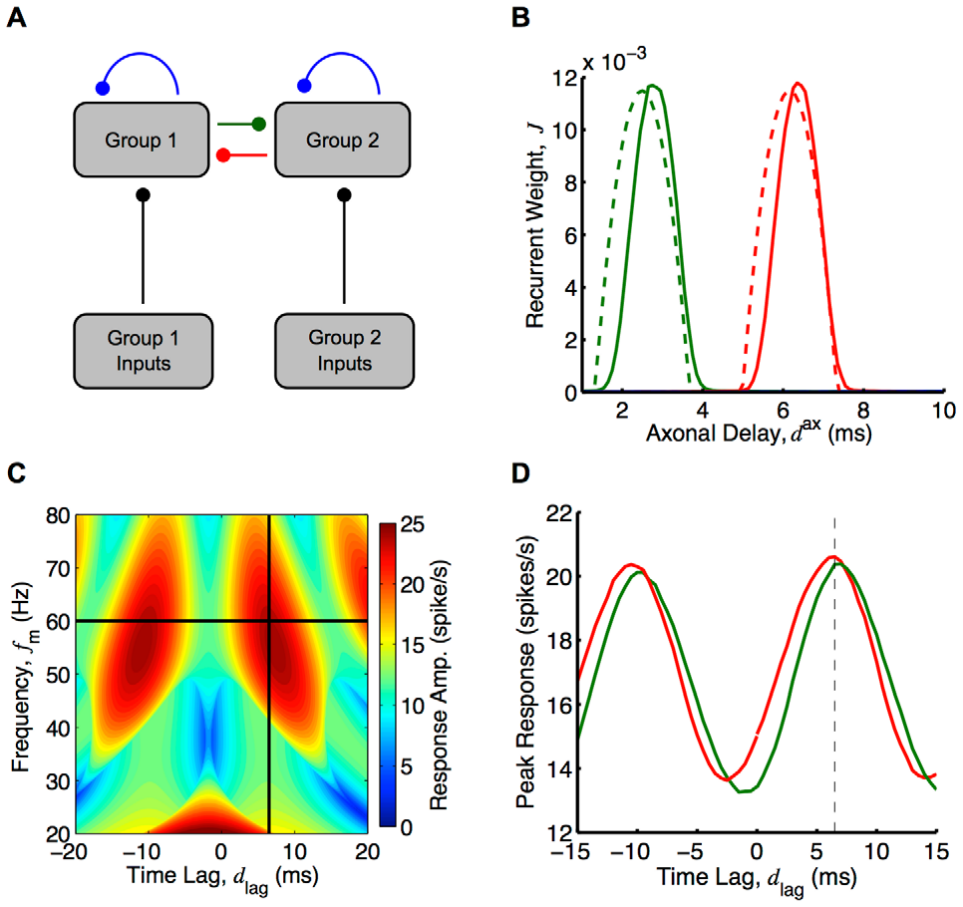
Axonal delay selection between two recurrently connected groups. (A) Diagram of two group model simplified from Figure 3. (B) Comparison between analytical (dashed) and simulation (solid) for axonal delay profiles between two groups each with oscillatory inputs with a modulation frequency of 60 Hz and where the inputs into group 2 are 6.5 ms behind the inputs into group 1. Analytical result was for 40,000s of learning and as given by Equation (45) and used a ‘slow’ EPSP. Simulation result was for 20,000s of learning with two groups of 5000 LIF neurons each. Shown are the delay profiles for the connections from group 2 to group 1 (red), from group 1 to group 2 (green), and within groups (blue) for which the mean weight for all delays was zero. (C) Analytically determined heat map (Equation (46)) of the mean of the group response amplitudes for different input frequencies and time lags. An EPSP with 1 ms rise time and 3 ms decay time was used instead of the ‘slow’ EPSP (Figure S6C), and recurrent strengths, 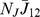 and 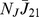, of 0.9. Black lines represent the training frequency (60 Hz) and time lag (6.5 ms). (D) Peak responses from simulations of group 1 (red) and group 2 (green) for different input time lags at the training frequency (60 Hz), for the network after learning, shown in B. Dashed vertical line represents training time tag. Both B, C and D use a modulation amplitude, 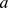, of 5 spikes/s, and the analytical plots in B and C used 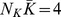, to match the network response to the network response during the simulation with the nonlinear LIF neurons.

We extended the learning of oscillatory inputs through axonal delay selection to learning within and between multiple groups of neurons. These groups each received oscillatory inputs of the same frequency but with different phases to each other. This extension of the model to two groups of neurons (and inputs) is described in the Methods section. In this case, we defined 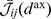 as the recurrent, weighted axonal delay profile for connections from group 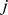 to group 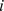. These sets of connections are shown in Figure 8A using different colors. This matrix of axonal delay profiles (specifically the deviation of these profiles from the homeostatic equilibrium) was predicted to evolve, due to STDP, according to
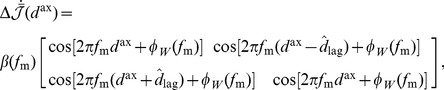
(45)where, as before, 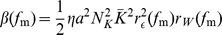.

An example of this delay selection at 60 Hz with a phase difference of 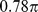 (6.5 ms) and ‘slow’ EPSPs (typical of pyramidal synapses in the cortex) is shown in Figure 8B. This shows the analytical prediction for the resulting delay profiles between the groups (red and green) after 25,000s of learning. It also shows the supporting simulations, which used two groups each of 5,000 LIF neurons. In both the analytical and simulation results, the two within-group axonal delay profiles are shown in blue but are not easily seen. This is because these connections were almost completely depressed and their plots lie close to the horizontal axis. We investigated analytically how the axonal delays that were selected between the groups depended on the time lag in Figure S5. For 60 Hz inputs, time lags of about 5 ms to 12 ms made it possible for the between-group connections to encode the frequency and time lag (for axonal delay ranges of 1–10 ms).

The response of each of the two groups, after delay selection, depended upon both the frequency of the inputs, 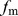, and the time lag between them, 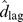 (their relative phases). As with a single group, the response of the groups was oscillatory with the same frequency as the inputs. We considered only the case where the within-group connections were completely depressed and played no role in the response. Given this, the amplitudes of the responses of groups 1 and 2, respectively, (derived in the Methods section) are approximated by
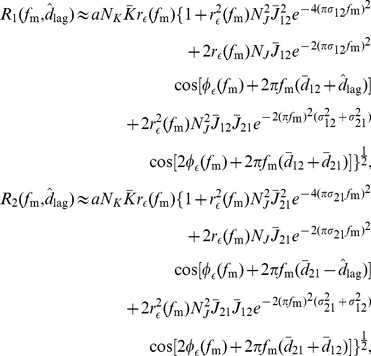
(46)where 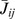, 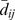, and 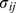 are the mean recurrent weight, and the mean and standard deviation (width) of the axonal delay profile, respectively, from group 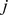 to group 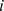.

Figure 8D shows the peak response of each group in the network from Figure 8B observed in simulations with various time lags between the inputs. Figure 8C shows the mean of the analytically determined group response amplitudes, 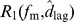 and 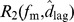, respectively, against both the input frequency, 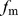, and the time lag between the inputs, 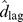. The individual group response amplitudes of this average are shown in Figure S6A and B. Both the analytical results and the simulations showed that the network response was selective to the trained time lag. For these results, an EPSP with a rise time of 1 ms and a decay time of 3 ms was used. This was in the parameter range between the ‘slow’ and ‘medium’ EPSPs, the results of which are shown in Figure S6C and D, respectively. The frequency that the network was predicted to be selective to differs from the training frequency, and these plots show that this difference strongly depended on the EPSP used. The strength of this selectivity, as with the response of a single group, depended on the mean strength of the recurrent connections. This is shown in Figure S6E, where the mean group response amplitudes are plotted for a network with weaker between group connections.

## Discussion

In this study, we examined how STDP can lead to the selection of axonal and dendritic propagation delays within a recurrently connected network receiving oscillatory synaptic input. We found that the recurrent delays selected depend on the frequency of the oscillations and that this delay selection influences the response of the network to different input frequencies. We identified the conditions under which the resulting network was selective to the frequency of oscillation that it received during learning.

For learning with only axonal delays (assuming short dendritic delays), the range of frequencies that can be learned is limited by the range of axonal delays in the network. This can be seen from Equation (42). Here, the maximum delay (10 ms in this study) sets the limit on the minimum frequency (76 Hz) and the minimum delay (1 ms) sets the limit on the maximum frequency (750 Hz).

After delay selection, the frequencies that a network can possibly respond selectively to differ from the frequencies that it can learn. While the minimum frequency is the same, the maximum frequency is limited by how reliably the neurons and synapses in the network respond to these higher frequencies. This is illustrated in Figure 6A and B. We also observed in Figure 6A that networks trained with higher frequencies (e.g. 240 Hz) have a higher response for low frequencies (e.g. 50 Hz). This is likely due to the width of the oscillation peaks at these low frequencies being much larger than potentiated axonal delays in the network. In this case, the oscillation peaks become reinforced by recurrent feedback multiple times during a single oscillation, increasing the response at lower frequencies.

We found that short dendritic delays are necessary in order for a network to become selective in its response to a particular frequency. This can be seen with a range of axonal and dendritic delays, where the connections that are selected during learning (red stripes in Figure 7) have a large range of total propagation delays (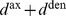). In other words, the learning is no longer a one-to-one mapping between frequency and delay. It has been suggested that, by considering unreliable synaptic transmission, STDP can differentiate between connections with the same difference in axonal and dendritic delays (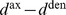) [33]. In this study, however, the effect of unreliable synaptic transmission is minimal because the post-synaptic activity arises predominately from the inputs rather than the recurrent connections. Without this one-to-one mapping between frequency and delay, the learned network is not selective to any frequency (let alone the training frequency). If dendritic delays are not short but are sharply tuned, the network would become selective to a frequency but it would not be the training frequency. In a network with a range of dendritic delays and short axonal delays, the network would also become frequency selective but again not to the training frequency. While there are unlikely to be regions in the brain that contain only connections with short dendritic delays, the sub-networks made up of only these short dendritic connections would exist. Depending on the relative strength and correlation of the inputs from connections with longer dendritic delays, the learning and response of the networks would be as we have considered here.

The STDP learning window used here has the typical bimodality (depression for post-pre spike pairs and potentiation for pre-post pairs) observed with excitatory synapses. The axonal delays of the recurrent connections temporally shift this learning window, and are potentiated if they do so by the right amount and capture a peak in the neurons' periodic correlogram. It is important to note that, because the oscillatory activity of the neurons is due to the inputs, coincidence detection by means of recurrent axonal delays is not necessary for learning. However, this recurrent coincidence detection is relevant when considering the amplitude of the network's response for different input frequencies.

As discussed in the Results section, recurrent excitatory networks have a critical point where the positive feedback of the potentiated recurrent connections causes run-away increases in firing. The mean strength of the recurrent connections converges to a homeostatic equilibrium that depends on the homeostatic learning parameters, the learning window, and the level of correlation in the inputs. This may be above or below this critical point. For networks where the mean recurrent weights tends to increase, Lubenov and Siapas showed that STDP keeps this mean weight just below the critical point, provided the connections have axonal delays that exceed their dendritic delays [36]. In this situation, synchronous firing is caused by the super-critical feedback. This synchronous firing is propagated along the recurrent axons arriving at neurons shortly after post-synaptic spikes, leading to synaptic depression. We observed this to be the case for the learning that was performed in this study (see Figure 4C). The networks taken after 20,000s of learning (see Figure 4D) all had mean recurrent weights just less than this critical value. We showed that the frequency selectively of the network increases dramatically as it approaches criticality.

We found our results to be robust with respect to the shape of the learning window used, provided it has the bimodality typical of excitatory synapses (see Figure S3). We also explored multiple neuron models (Poisson and LIF) and a range of synaptic time constants (‘slow’, ‘medium’ and ‘fast’ EPSPs). The delay selection learning within a single group requires a significantly large number of neurons in the network (see Section 1 of Supporting Text S1 and Figure S7A). In spiking networks, a particular synaptic input may be the input that induces an output spike, which is referred to as a spike triggered correlation [8], [17]. In the Poisson neuron model, this is captured by the autocorrelation of the external inputs and the autocorrelation of the neurons in the network. This effect is small if many inputs are required for a neuron to generate a spike. If the network is too small, these correlations dominate the shape of the delay profile learned and prevent the encoding of the input frequency. For axonal delay selection between groups, where the spike triggering correlations are not present, smaller numbers of neurons (e.g. 1000) can demonstrate axonal delay selection than is possible within a single group (see Figure S7B).

This study focused on additive STDP and more general STDP models remain to be fully explored [37]. Although not investigated here, we would expect a weight dependent (non-additive) learning rule to produce qualitatively similar results as long as the weight dependency leads to sufficient competition between the recurrent weights [12], [18], [38], [39]. Another variant of STDP that was not considered here is triplet STDP [40]–[43]. Triplet STDP effectively modifies the learning window for different firing rates and captures correlations beyond the second order [44]. The correlations in our model arise solely from oscillations and there are no higher-than-second-order correlations (see Section 8 of Supporting Text S1). Also, our results have been shown to be reasonably insensitive to the precise shape of the typical excitatory STDP learning window for the frequency range considered here (see Figure S3). Therefore, we would expect qualitatively similar results with a triplet STDP model, provided that the mean firing rates are in the range such that there is both LTP and LTD (approximately 5–30 Hz [44]).

In this study, we looked only at the learning of oscillatory (sinusoidal) patterns. However, this delay selection learning could potentially encode many other temporal patterns. This study suggests the ranges of the frequency spectra of the types of signals that could be learned. A slightly different pattern to consider would be a periodic, phase-locked firing pattern. Here, the firing rate would be made up of a small base level with narrow, high intensity peaks that periodically occur. In this situation, we would expect delay selection to occur in a manner similar to that described in this study, or possibly faster. Trained networks would be expected to respond less to low frequencies (much lower than the training frequency). This is because the narrow peaks within the inputs would not allow the behavior observed in this study, where recurrent connections reinforced activity within the same oscillation peak (see Figure 6A).

Delay selection can also encode the oscillation frequency of signals in the feed-forward connection delays [5]. This requires selecting two (or more) different delays, the difference of which would need to be a multiple of the oscillation period. Depending on the frequency, this may require an unrealistic range of delays in the connections, especially if these are connections within a local network.

**Figure 9:**
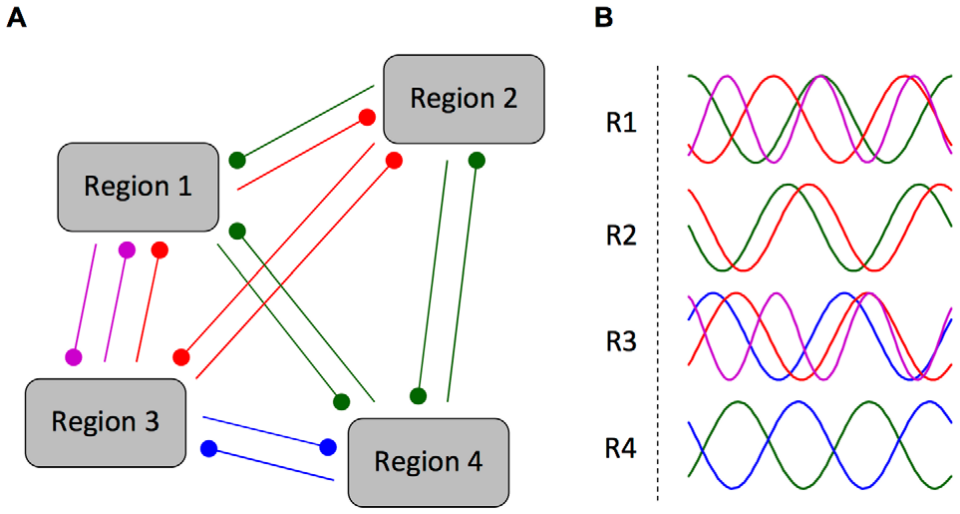
Illustration of different neuronal ensembles formed by delay selection. (A) Multiple groups/regions of recurrently connected neurons where connections between them have undergone axonal delay selection appropriate to the different out-of-phase, oscillatory activities present during the activation of each neuronal ensemble (blue, red, green and magenta). (B) Different out-of-phase, oscillatory activity of each group/region for the different neuronal ensembles (blue, red, green and magenta). In this network, only one neuronal ensemble can ever be ‘active’ at once. For each neuronal ensemble, the activities of the participating regions have the specific frequencies and relative phases that drove the delay selection and, after learning, resonate with the selected axonal delays of the recurrent connections.

First proposed by Hebb [45], there has been growing experimental evidence for spatially distributed neuronal ensembles or cell assemblies [46]–[49] in which neurons far apart in the brain are synchronously active. This type of coordinated activity between distant regions (or cortical ‘columns’ in these regions) may be mediated by long range synaptic connections. The learning considered in this study, where delay selection is observed between multiple groups that are driven by out-of-phase, gamma oscillations, provides a possible mechanism by which this type of behavior could arise. Gamma (and other cortical) oscillations are known to be stronger during certain cognitive tasks [27]. This strengthening may correspond to the “activation” of particular ensembles between certain regions/columns, as illustrated in Figure 9A. These “activations”, as described here, would be triggered by specific sets of phase differences as shown in Figure 9B. This would be similar to proposed gating [50] and routing mechanisms [51]. If the learning we have considered here does lead to the formation of these types of neuronal ensembles, then it can be seen that it would be possible for these ensembles to merge with one another, split into multiple ensembles, grow in size, shrink, temporally bind with one another, or trigger, promote, stop, or block one another. Such ensemble activity is thus related to studies on neural syntax [52], neural Darwinism [53], synfire chains [54], and polychronous groups [10] (although at the larger scale of regions/columns than polychronous groups).

This study focused on the oscillatory activity in the network being due to oscillations in the inputs. These inputs may represent activity coming from sensory areas and representing a stimulus (e.g. sound), but they may also be involved in the coding of information coming from other brain regions. In this case, the oscillations would not necessarily represent the oscillatory nature of a stimulus, but instead have some functional role (such as the above mentioned cognitive role postulated for gamma oscillations). The oscillations in the networks may even be intrinsically generated and not due to the inputs at all. In a related study, Cateau et. al. looked at the oscillations that arose in a network due to STDP and the specific properties of the neuron model used [55]. They observed functional clustering of neurons into groups with only weak within group connections. Since there was only a narrow range of axonal delays in the study, STDP was not able to perform delay selection. The study demonstrated that STDP instead selected the number of out-of-phase groups of neurons such that the connections between the groups all “shared” the oscillation delay.

Pyramidal-interneuron gamma (PING) is an established network configuration that generates intrinsic oscillations via the interactions between excitatory and inhibitory populations [56], [57]. If it is assumed that inhibitory connections in the cortex only act locally, local populations producing these oscillations would be connected to other local populations by only excitatory connections. These interconnected groups are similar to those considered in this study. The main difference is that their oscillations are internally generated rather than due to inputs. Though it remains to be considered, we would expect the synaptic changes to the excitatory connections between these groups to occur in a qualitatively similar manner to what was observed between the multiple groups considered in this paper. Furthermore, when oscillations are internally generated, the phase of their oscillations is not fixed and can be perturbed. In this situation, it is possible that the delay selection between multiple groups could lead to the formation of stable patterns of relative phases or oscillatory states or modes. Although the phase of the oscillations is not fixed, the frequency of the oscillations would be fixed at a frequency determined by the excitatory-inhibitory interactions. Because of this, the frequency selectivity of the network response due to the delay selection would not be of interest. However, the phase selectivity of the network response would be relevant. We would expect a stronger response when the activity is in one of the stable oscillatory modes. Inputs to the network (not necessarily oscillatory) could possibly steer the network in and out of these different, self-sustaining modes. This formation of neuronal ensembles from intrinsic network oscillations may even be possible with multiple, distinct oscillation frequencies present (e.g. gamma and beta frequencies) [58], [59]. This suggests another extension for the learning between multiple groups, which only considered a single frequency. Investigating how these networks could encode two (or more) different frequencies, instead of a single frequency and a time lag, is left as an interesting challenge.

Another example of where the delay selection mechanism might be employed in the brain is in the auditory brainstem. Here, sound information is encoded both spatially, with different frequency components being carried by spike trains of different nerve fibers, and temporally, with the precise timing of these spikes. The delay selection mechanism considered in this study may provide a way to extract the temporal information. Specifically, it could help explain how the brain can perceive the pitch of a sound with a missing fundamental, such as in telephone speech. The frequency range we have considered (100–300 Hz) is typical of the pitches present in speech. The neuronal and synaptic time constants that we have used (‘fast’ EPSP) are consistent with those observed in the auditory brainstem. We demonstrated how oscillations in this range can be encoded into the axonal delays of a network, which becomes selectively responsive to this trained frequency. It remains to be explored whether networks could be trained instead with a complex sound containing a fundamental frequency (e.g. 120 Hz) as well as its harmonics (e.g. 240, 360, and 480 Hz). It would then be of interest whether the network became selective not only to this signal but also to the corresponding signal without the fundamental frequency. If this were shown with a sufficiently detailed simulation with realistic inputs, this mechanism would be a candidate for describing missing fundamental pitch perception. However, other possible mechanisms are likely to exist and these would each need to be tested against experimental data.

Throughout this study, we only considered networks of excitatory neurons. This is an important point to note because, as mentioned, studies have shown that interactions between excitatory and inhibitory populations can lead to the generation of intrinsic oscillations (e.g. PING) [56], [57]. Furthermore, the networks considered all operated in super-threshold, mean-driven regimes. This was done to facilitate the mathematical analysis and reduce the number of parameters to consider. The present analytic framework has not been extended to incorporate inhibitory post-synaptic potentials and this could be an area for future investigation. The current model provides a suitable description of the feed-forward behavior of neural processing in the auditory brainstem (for a review, see [60]). Conversely, the cortex is generally considered to be a balanced network, operating in a fluctuation-driven regime [61]–[67]. Because of this, it is not clear how applicable the results in this study are to such networks. However, as we discussed, our results for multiple, out-of-phase groups would be expected to extend to the situation where local populations of excitatory and inhibitory neurons, internally generating (gamma) oscillations (through a mechanism such as PING) are connected to each other through long-range, excitatory connections. This situation may provide a more suitable model of the cortex [68], [69].

STDP in a network regularly driven by oscillatory activity introduces a bias towards strong connections with a specific linear relationship between their axonal and dendritic delays, as shown in Figure 7. If a structural plasticity mechanism exists in the brain that physically removes weak connections not being used, then this predicts that a bias should be observed in the axonal and dendritic delays of connections in regions of the brain known to exhibit oscillatory activity. For example, the relationship between these delays shown in Figure 7B may be what is observed in regions of the cortex that show gamma oscillations. This prediction assumes that STDP works in the same manner for connections with long dendritic delays. While it is usually modeled in this way [37], it is not clear whether this is consistent with experimental work [70], [71].

## Supporting Information

Figure S1
**Amplitude of the Fourier transform of the learning window as a function of frequency.** Given by Equations (31) in Section 6 of Supporting Text S1. The standard window has parameters 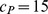, 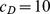, 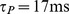 and 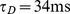.(TIFF)

Figure S2 **Comparison of learning predicted analytically (dashed) and from a simulation with Poisson neurons (solid).** The axonal delay profile of a network at homeostatic equilibrium with 120 Hz oscillatory inputs after 1000s (blue), 2000s (green), 3000s (red), 4000s (cyan), 5000s (magenta), 6000s (yellow), and 7000s (black). A ‘medium’ EPSP was used here.
(TIFF)

Figure S3
**Analytical comparison of frequency correspondence between learning and response for different learning windows.** Plot of the training frequency and corresponding peak response frequency for different learning windows for a ‘fast’ EPSP, a delay profile with width, 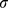, of 0.5 ms, strength, 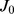, of 0.5, and with a modulation amplitude, 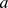, of 5 spikes/s. The different learning windows shown are: the standard window with 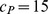, 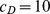, 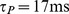 and 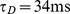 (green), the standard window with 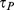 and 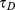 multiplied by 0.1 (blue), the standard window with 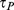 and 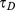 multiplied by 10 (red), a balanced window with 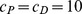 and 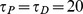 (magenta), and a window biased in the reverse way to the standard with 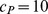, 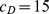, 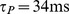 and 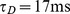 (yellow). The dashed line represents 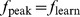.(TIFF)

Figure S4 **Comparison of analytical expression for network response with simulations using the Poisson neuron model.** (A) The axonal delay profile of a network before (green) and after (blue) 40,000s of learning, with STDP and 120 Hz inputs and a Gaussian delay profile (red), which closely approximates the profile after learning. (B) The amplitude of the network response to different input modulation frequencies, from simulations with networks of the same color in A (green and blue), or analytically determined from the Gaussian delay profile of the same color in A (red), using Equation (43). A ‘medium’ EPSP was used here.
(TIFF)

Figure S5 **Selected axonal delays for different time lags between the inputs into two groups.** (A) Analytical plot of the axonal delays selected by STDP for connections within each of two groups (blue), from group 1 to group 2 (green), and from group 2 to group 1 (red), with the time lag between the 60 Hz oscillatory inputs into each group. The dashed line represents the 6.5 ms time lag considered in more detail. (B) Same as A (thick lines) with additional lines for 55 Hz (paler lines) and 65 Hz (darker lines). Note that the three green lines (pale, thick and dark) in the bottom right of B are very close together.
(TIFF)

Figure S6
**Analytical estimations of two group response amplitude to different inputs.** (A) Response amplitude of group 1 for inputs with different frequencies and relative time lags, using Equation (46) with an EPSP with a rise time of 1 ms and a decay time of 3 ms, a modulation amplitude, 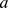, of 5 spikes/s, feedforward strengths, 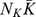, of 1.0, and recurrent strengths, 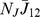 and 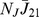, of 0.9. (B) Same as A but for group 2. (C) Plot of the average between the response amplitudes of groups 1 and 2 as plotted for A and B but with a ‘slow’ EPSP. (D) Same as C but with a ‘medium’ EPSP. (E) Same as C but with the EPSP used in A and B and weaker recurrent strengths of 0.5.(TIFF)

Figure S7 **Axonal delay profile learned in networks of different sizes.** (A) Deviation of axonal delay profile (from mean network weight) after 250s of learning for a single group of 500 (blue), 1000 (red), 2000 (green), 5000 (magenta), 10,000 (cyan), 20,000 (black, dashed) LIF neurons receiving 120 Hz inputs and with ‘medium’ EPSPs. (B) Deviation of axonal delay profile (from mean network weight), for connections from group 2 to group 1, after 100s of learning for two groups, each with 250 (blue), 500 (red), 1000 (green), 2000 (magenta), 5000 (cyan), and 10,000 (black, dashed) LIF neurons, receiving different, out-of-phase (6.5 ms), 60 Hz inputs and with ‘slow’ EPSPs.
(TIFF)

Text S1 **Detailed analytical derivations and calculations.** (PDF). Sections: (1) Recurrent Correlation, (2) Oscillatory Inputs, (3) Homeostatic Equilibrium in a Recurrent Network, (4) Network Response for a Single Group, (5) Network Response for Two Groups, (6) Learning Window and EPSP Kernel, (7) Estimating the Amplitude of a Sum of Cosines, and (8) Third-Order Covariance of Oscillatory Inputs.
(PDF)
