# Supplementary figures and images for: Delay Selection by Spike-Timing-Dependent Plasticity in Recurrent Networks of Spiking Neurons Receiving Oscillatory Inputs
Source: PLoS Comput Biol. 2013 Feb 7;9(2):e1002897. doi: 10.1371/journal.pcbi.1002897 (PMC3567188; doi:10.1371/journal.pcbi.1002897)

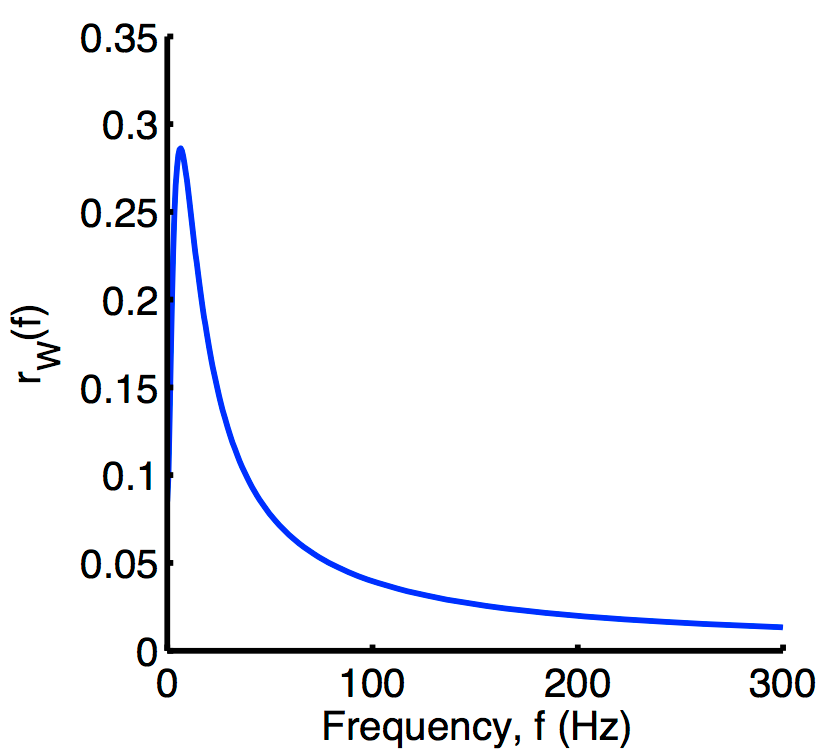

Supplement: Figure S1 — Amplitude of the Fourier transform of the learning window as a function of frequency. Given by Equations (31) in Section 6 of Supporting Text S1. The standard window has parameters , , and . (TIFF) [file pcbi.1002897.s001.tiff]

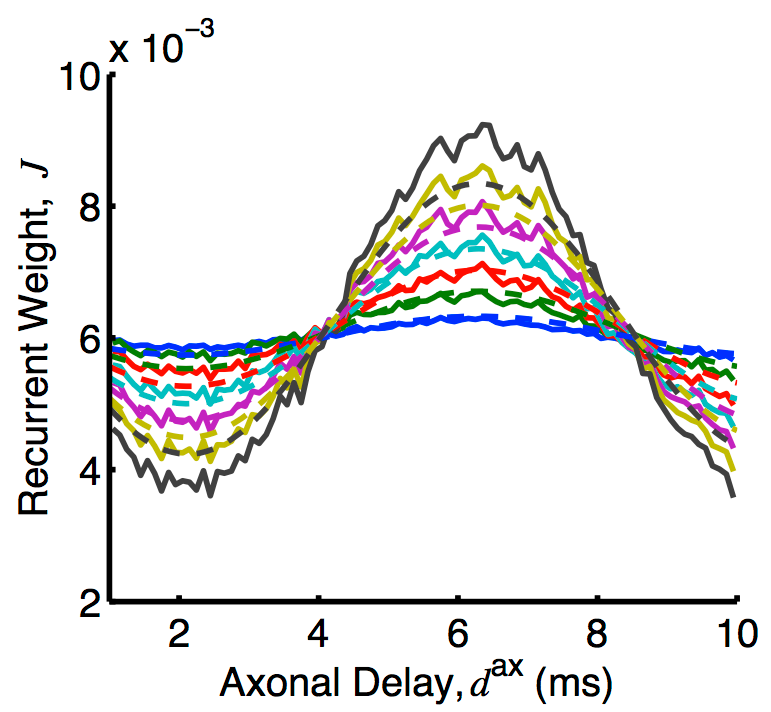

Supplement: Figure S2 — Comparison of learning predicted analytically (dashed) and from a simulation with Poisson neurons (solid). The axonal delay profile of a network at homeostatic equilibrium with 120 Hz oscillatory inputs after 1000s (blue), 2000s (green), 3000s (red), 4000s (cyan), 5000s (magenta), 6000s (yellow), and 7000s (black). A ‘medium’ EPSP was used here. (TIFF) [file pcbi.1002897.s002.tiff]

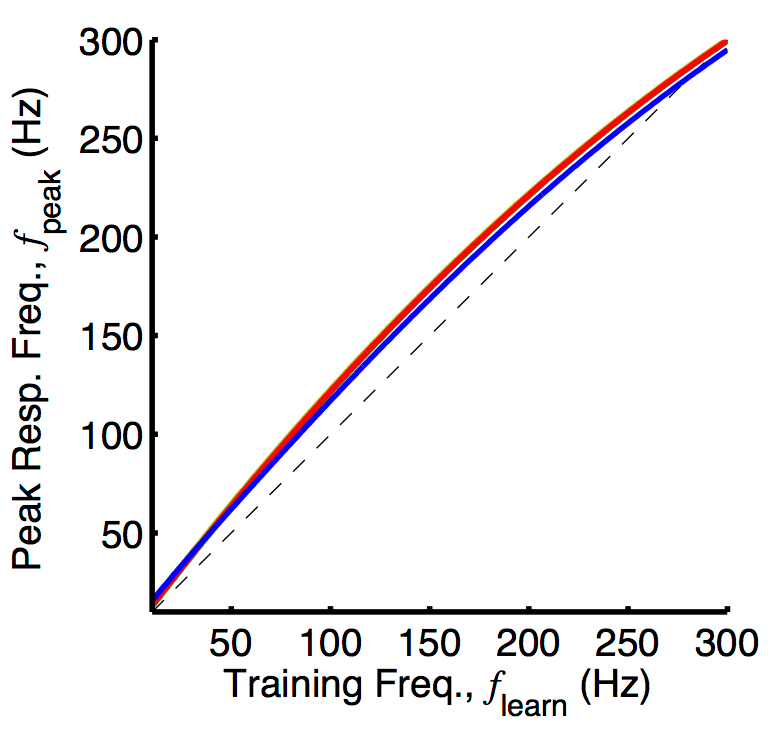

Supplement: Figure S3 — Analytical comparison of frequency correspondence between learning and response for different learning windows. Plot of the training frequency and corresponding peak response frequency for different learning windows for a ‘fast’ EPSP, a delay profile with width, , of 0.5 ms, strength, , of 0.5, and with a modulation amplitude, , of 5 spikes/s. The different learning windows shown are: the standard window with , , and (green), the standard window with and multiplied by 0.1 (blue), the standard window with and multiplied by 10 (red), a balanced window with and (magenta), and a window biased in the reverse way to the standard with , , and (yellow). The dashed line represents . (TIFF) [file pcbi.1002897.s003.tiff]

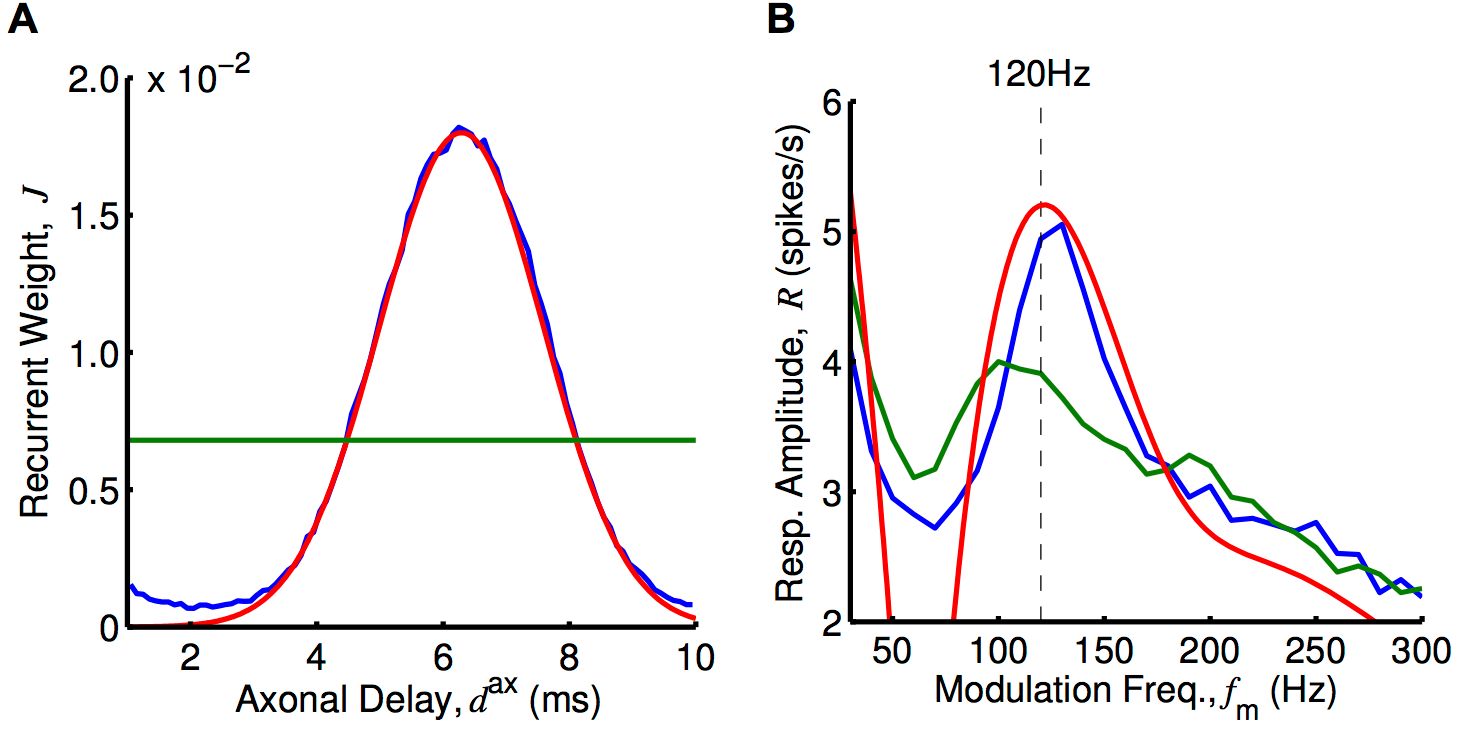

Supplement: Figure S4 — Comparison of analytical expression for network response with simulations using the Poisson neuron model. (A) The axonal delay profile of a network before (green) and after (blue) 40,000s of learning, with STDP and 120 Hz inputs and a Gaussian delay profile (red), which closely approximates the profile after learning. (B) The amplitude of the network response to different input modulation frequencies, from simulations with networks of the same color in A (green and blue), or analytically determined from the Gaussian delay profile of the same color in A (red), using Equation (43). A ‘medium’ EPSP was used here. (TIFF) [file pcbi.1002897.s004.tiff]

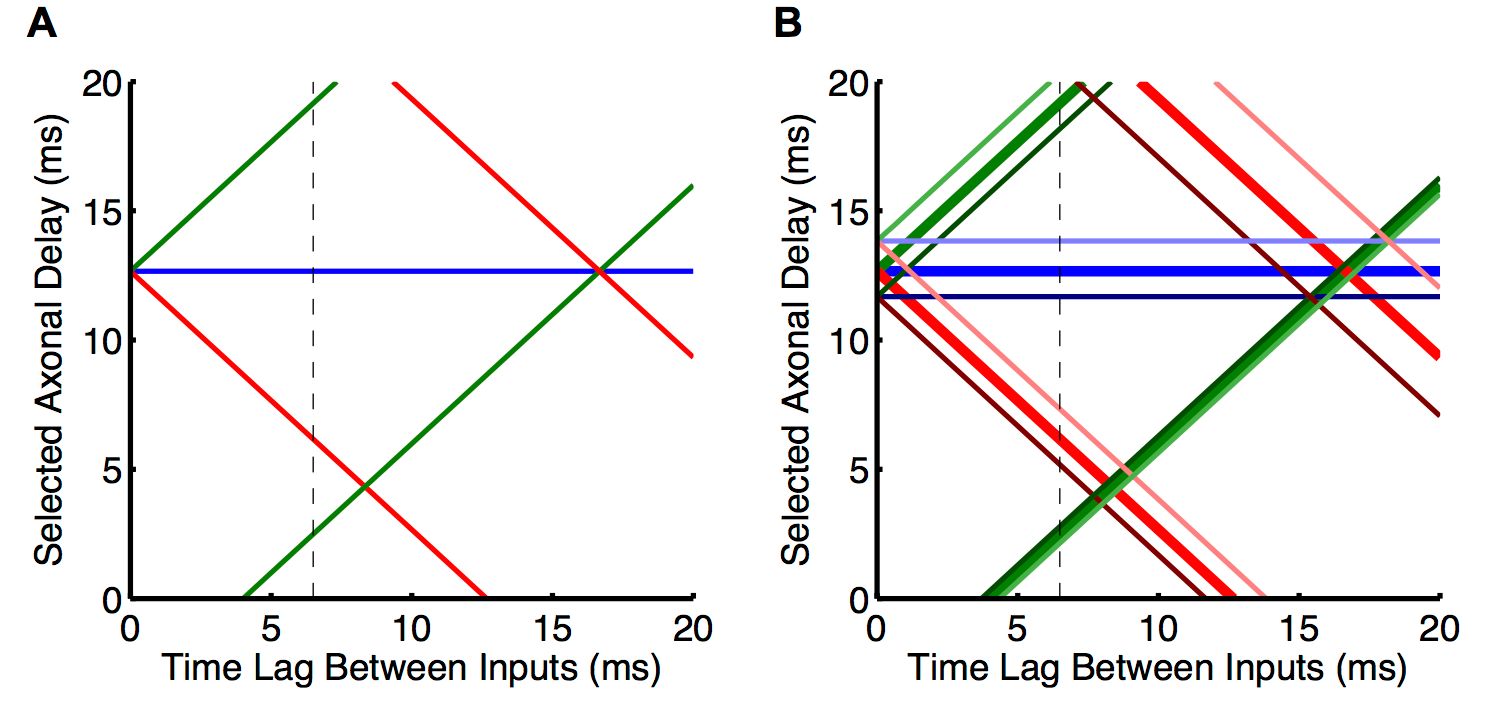

Supplement: Figure S5 — Selected axonal delays for different time lags between the inputs into two groups. (A) Analytical plot of the axonal delays selected by STDP for connections within each of two groups (blue), from group 1 to group 2 (green), and from group 2 to group 1 (red), with the time lag between the 60 Hz oscillatory inputs into each group. The dashed line represents the 6.5 ms time lag considered in more detail. (B) Same as A (thick lines) with additional lines for 55 Hz (paler lines) and 65 Hz (darker lines). Note that the three green lines (pale, thick and dark) in the bottom right of B are very close together. (TIFF) [file pcbi.1002897.s005.tiff]

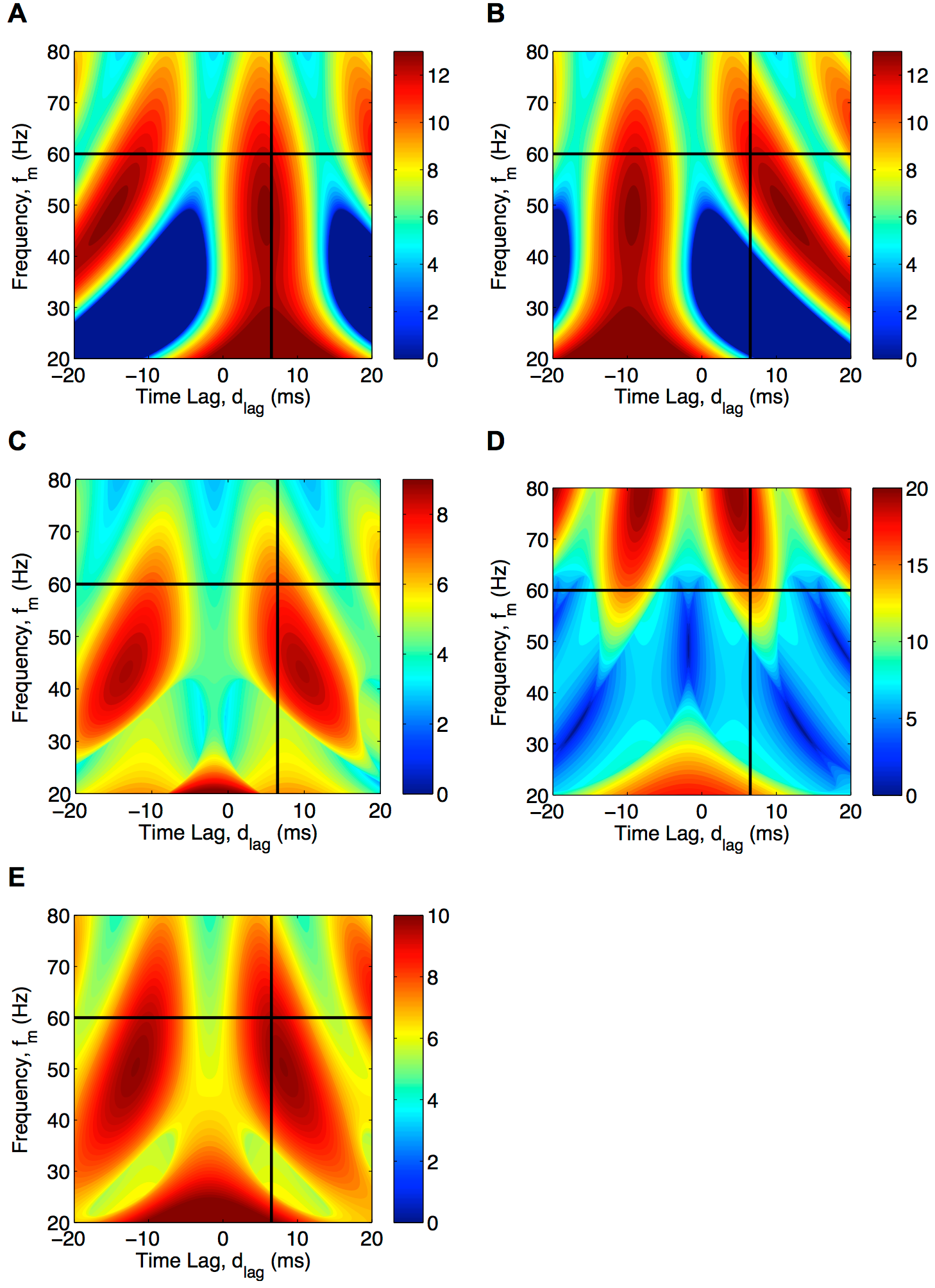

Supplement: Figure S6 — Analytical estimations of two group response amplitude to different inputs. (A) Response amplitude of group 1 for inputs with different frequencies and relative time lags, using Equation (46) with an EPSP with a rise time of 1 ms and a decay time of 3 ms, a modulation amplitude, , of 5 spikes/s, feedforward strengths, , of 1.0, and recurrent strengths, and , of 0.9. (B) Same as A but for group 2. (C) Plot of the average between the response amplitudes of groups 1 and 2 as plotted for A and B but with a ‘slow’ EPSP. (D) Same as C but with a ‘medium’ EPSP. (E) Same as C but with the EPSP used in A and B and weaker recurrent strengths of 0.5. (TIFF) [file pcbi.1002897.s006.tiff]

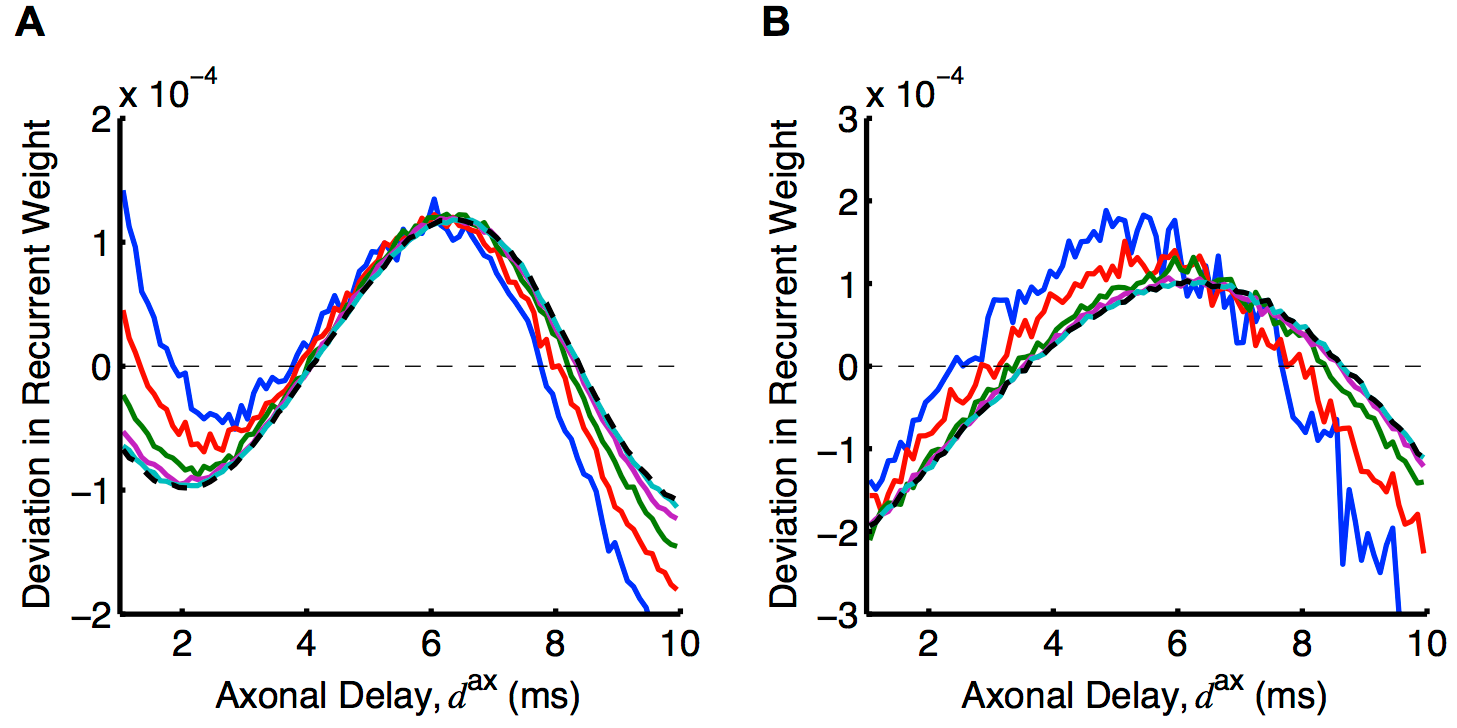

Supplement: Figure S7 — Axonal delay profile learned in networks of different sizes. (A) Deviation of axonal delay profile (from mean network weight) after 250s of learning for a single group of 500 (blue), 1000 (red), 2000 (green), 5000 (magenta), 10,000 (cyan), 20,000 (black, dashed) LIF neurons receiving 120 Hz inputs and with ‘medium’ EPSPs. (B) Deviation of axonal delay profile (from mean network weight), for connections from group 2 to group 1, after 100s of learning for two groups, each with 250 (blue), 500 (red), 1000 (green), 2000 (magenta), 5000 (cyan), and 10,000 (black, dashed) LIF neurons, receiving different, out-of-phase (6.5 ms), 60 Hz inputs and with ‘slow’ EPSPs. (TIFF) [file pcbi.1002897.s007.tiff]
