# Supplementary material for: Delay Selection by Spike-Timing-Dependent Plasticity in Recurrent Networks of Spiking Neurons Receiving Oscillatory Inputs
Source: PLoS Comput Biol. 2013 Feb 7;9(2):e1002897. doi: 10.1371/journal.pcbi.1002897 (PMC3567188; doi:10.1371/journal.pcbi.1002897)
Supplement: Text S1 — Detailed analytical derivations and calculations. (PDF). Sections: (1) Recurrent Correlation, (2) Oscillatory Inputs, (3) Homeostatic Equilibrium in a Recurrent Network, (4) Network Response for a Single Group, (5) Network Response for Two Groups, (6) Learning Window and EPSP Kernel, (7) Estimating the Amplitude of a Sum of Cosines, and (8) Third-Order Covariance of Oscillatory Inputs. (PDF) [file pcbi.1002897.s008.pdf]

Supporting Information: Text S1

1 Recurrent Correlation

As given by Equation (58) of [1], the (ordinary frequency) Fourier transform, $\mathcal{F}g(f) = \int_{-\infty}^{\infty} g(x)e^{-2\pi ixf}dx$, of the recurrent correlation function for a network with only axonal delays is

$$\mathcal{F}C(f) = Q(f)\Big\{P(f)\big[\mathcal{F}\hat{C}(f) + \mathrm{diag}(\hat{\nu})\big]P^T(-f) + \mathrm{diag}(\nu)\Big\}Q^T(-f) - \mathrm{diag}(\nu), \tag{1}$$

where

$$\begin{aligned} Q_{jk}(f) &= [I - J_{jk}e^{2\pi i d^{\mathrm{ax}}_{jk} f}\mathcal{F}\epsilon(-f)]^{-1}, \\ P_{jk}(f) &= K_{jk}e^{2\pi i \hat{d}^{\mathrm{ax}}_{jk} f}\mathcal{F}\epsilon(-f). \end{aligned} \tag{2}$$

It can be considered be to make up of three components

$$\begin{aligned} C(u) &= C_1(u) + C_2(u) + C_3(u) \\ \mathcal{F}C(f) &= \mathcal{F}C_1(f) + \mathcal{F}C_2(f) + \mathcal{F}C_3(f), \end{aligned} \tag{3}$$

where

$$\begin{aligned} \mathcal{F}C_1(f) &= Q(f)P(f)\mathcal{F}\hat{C}(f)P^T(-f)Q^T(-f), \\ \mathcal{F}C_2(f) &= Q(f)P(f)\mathrm{diag}(\hat{\nu})P^T(-f)Q^T(-f), \\ \mathcal{F}C_3(f) &= Q(f)\mathrm{diag}(\nu)Q^T(-f) - \mathrm{diag}(\nu). \end{aligned} \tag{4}$$

These components are due to correlations in the inputs, spike triggering effects from the inputs, and recurrent spike triggering effects, respectively. The last two of these are assumed to be negligible to the learning for large numbers of inputs, M, and large numbers of neurons, N, respectively. This is the same assumption made in [1]. Because of this only the first correlation component was considered (i.e. $\mathcal{F}\bar{C}(f) \approx \mathcal{F}\bar{C}_1(f)$).

To determine how large a network was sufficient for the spike triggering components to be negligible, simulations with LIF neurons were run to observe the shape of the learned axonal delay distribution after 250s of learning. This is shown in Figure S7. For simulations it was decided that the network size would always be the same as the number of inputs (i.e. $N = M$). It can be seen that as the number of neurons (and inputs) increases, the resulting delay distribution becomes a perfect cosine function. We decided that 10,000 neurons (and inputs) was sufficient for simulations in this study.

2 Oscillatory Inputs

Input intensity functions are defined for oscillatory inputs as

$$\hat{\lambda}_k(t) = \langle \hat{S}_k(t)\rangle = \hat{\nu}_0 + a\cos\big[2\pi f_m(t + \hat{d}_k)\big], \tag{5}$$

where $\hat{\nu}_0$ is the mean input rate (in Hz), a is the magnitude of the oscillations (in Hz), f_m is the modulation frequency of the oscillations (in Hz), and $\hat{d}_k$ is the delay of the input (in seconds). Inputs within the same group have the same delay, meaning that they are in phase.

The mean input firing rate of neuron k is

$$\begin{aligned} \hat{\nu}_k &= \frac{1}{T}\int_{t-T}^{t} \langle \hat{S}_k(t')\rangle dt' = \frac{1}{T}\int_{t-T}^{t} \Big\{\hat{\nu}_0 + a\cos\big[2\pi f_m(t + \hat{d}_k)\big]\Big\}dt' \\ &= \hat{\nu}_0 + \frac{a}{T}\int_{t-T}^{t} \cos\big[2\pi f_m(t + \hat{d}_k)\big]dt' = \hat{\nu}_0. \end{aligned} \tag{6}$$

The correlation function for a pair of inputs (k and l) is

$$\begin{aligned}
\hat{C}_{kl}(t,u) &= \frac{1}{T}\int_{t-T}^{t}\langle \hat{S}_k(t')\hat{S}_l(t'+u)\rangle dt' - \left(\frac{1}{T}\int_{t-T}^{t}\langle \hat{S}_k(t')\rangle dt'\right)\left(\frac{1}{T}\int_{t-T}^{t}\langle \hat{S}_l(t'+u)\rangle dt'\right) \\
&= \frac{1}{T}\int_{t-T}^{t}\Big\{\hat{\nu}_0 + a\cos\big[2\pi f_m(t'+\hat{d}_k)\big]\Big\}\Big\{\hat{\nu}_0 + a\cos\big[2\pi f_m(t'+u+\hat{d}_l)\big]\Big\}dt' - \hat{\nu}_0^2 \\
&= \hat{\nu}_0^2 + \frac{\hat{\nu}_0 a}{T}\int_{t-T}^{t}\Big\{\cos\big[2\pi f_m t'\big] + \cos\big[2\pi f_m(t'+u+\hat{d}_{\text{lag}})\big]\Big\}dt' \\
&\quad + \frac{a^2}{T}\int_{t-T}^{t}\cos\big[2\pi f_m t'\big]\cos\big[2\pi f_m(t'+u+\hat{d}_{\text{lag}})\big]dt' - \hat{\nu}_0^2 \\
&= \frac{a^2}{2T}\int_{t-T}^{t}\Big\{\cos\big[2\pi f_m(u+\hat{d}_{\text{lag}})\big] + \cos\big[2\pi f_m(2t'+u+\hat{d}_{\text{lag}})\big]\Big\}dt' \\
&= \frac{a^2}{2}\cos\big[2\pi f_m(u+\hat{d}_{\text{lag}})\big],
\end{aligned} \tag{7}$$

where $\hat{d}_{\text{lag}} = \hat{d}_l - \hat{d}_k$, and the Fourier transform of this is

$$\mathcal{F}\hat{C}_{kl}(f) = \frac{a^2}{4}\Big[\delta(f-f_m) + \delta(f+f_m)\Big]e^{2\pi i\hat{d}_{\text{lag}}f}. \tag{8}$$

If the inputs are from the same group, then $\hat{d}_{\text{lag}} = 0$, and so

$$\begin{aligned}
\hat{C}_{kl}(u) &= \frac{a^2}{2}\cos\big(2\pi f_m u\big), \\
\mathcal{F}\hat{C}_{kl}(f) &= \frac{a^2}{4}\Big[\delta(f-f_m) + \delta(f+f_m)\Big].
\end{aligned} \tag{9}$$

3 Homeostatic Equilibrium in a Recurrent Network

The rate of change of the recurrent axonal delay distribution is

$$\dot{\bar{\mathcal{J}}}(t,d^{\text{ax}}) = \eta\Big[\omega_{\text{in}}\bar{\nu}(t) + \omega_{\text{out}}\bar{\nu}(t) + \tilde{W}\bar{\nu}(t)^2 + \bar{C}^W(t,d^{\text{ax}})\Big], \tag{10}$$

where $\bar{\nu}(t)$ is the mean firing rate of the recurrent group given by

$$\begin{aligned}
\bar{\nu} &= \frac{\nu_0 + N_K\bar{K}\hat{\nu}_0}{1 - \mathcal{F}\epsilon(0)\tilde{N}_J\mathcal{F}\bar{\mathcal{J}}(0)} \\
&= \frac{\nu_0 + N_K\bar{K}\hat{\nu}_0}{1 - \tilde{N}_J\int_{d_{\min}}^{d_{\max}}\bar{\mathcal{J}}(x)dx} \\
&= \frac{\nu_0 + N_K\bar{K}\hat{\nu}_0}{1 - N_J\bar{J}},
\end{aligned} \tag{11}$$

where ν_0 is the spontaneous firing rate of the neurons, $\hat{\nu}_0$ is the mean firing rate of the inputs, and $\bar{J}$ is the mean recurrent weight averaged over all axonal delays. The stable mean firing rate, $\bar{\nu}^*$, and stable mean weight, $\bar{J}^*$, are found from

$$\begin{aligned}
\dot{\bar{J}} &\propto (\omega_{\text{in}} + \omega_{\text{out}})\bar{\nu} + \tilde{W}\bar{\nu}^2 + \int_{d_{\min}}^{d_{\max}}\bar{C}^W(x)dx \\
0 &= (\omega_{\text{in}} + \omega_{\text{out}})\bar{\nu}^* + \tilde{W}(\bar{\nu}^*)^2 + \bar{C}^W.
\end{aligned} \tag{12}$$

Assuming $\bar{C}^W$ is small and that $\nu_0 = 0$, the solution to this is

$$\bar{\nu}^* = \frac{-(\omega_{\text{in}} + \omega_{\text{out}})}{\tilde{W}}, \tag{13}$$

and, by substituting in Equation (11) from this supporting text, we have that

$$\begin{aligned} 1 - N_J \bar{J}^* &= \frac{N_K \bar{K} \hat{\nu}_0}{\bar{\nu}^*} = \frac{N_K \bar{K} \hat{\nu}_0 \tilde{W}}{-(\omega_{\text{in}} + \omega_{\text{out}})} \\ \bar{J}^* &= \frac{1}{N_J}\left(1 + \frac{N_K \bar{K} \hat{\nu}_0 \tilde{W}}{\omega_{\text{in}} + \omega_{\text{out}}}\right). \end{aligned} \tag{14}$$

4 Network Response for a Single Group

Given the average response is

$$\bar{\lambda}(t) = \tilde{N}_J \int_{d_{\min}}^{d_{\max}} \bar{\mathcal{J}}(x) \int \epsilon(r-x)\bar{\lambda}(t-r)drdx + N_K \bar{K} \int \epsilon(r-\hat{d})\hat{\lambda}(t-r)dr, \tag{15}$$

where $\hat{d}$ is the delay of the inputs. The Fourier transform of this is

$$\mathcal{F}\bar{\lambda}(f) = \tilde{N}_J \mathcal{F}\bar{\mathcal{J}}(f)\mathcal{F}\epsilon(f)\mathcal{F}\bar{\lambda}(f) + N_K \bar{K} e^{-2\pi i \hat{d} f}\mathcal{F}\epsilon(f)\mathcal{F}\hat{\lambda}(f), \tag{16}$$

and by rearranging this we get

$$\mathcal{F}\bar{\lambda}(f) = \frac{N_K \bar{K} e^{-2\pi i \hat{d} f}\mathcal{F}\epsilon(f)\mathcal{F}\hat{\lambda}(f)}{1 - \tilde{N}_J \mathcal{F}\bar{\mathcal{J}}(f)\mathcal{F}\epsilon(f)}. \tag{17}$$

For oscillatory inputs where $\hat{\lambda}(t) = \hat{\nu}_0 + a\cos(2\pi f_m t)$ and $\mathcal{F}\hat{\lambda}(f) = \hat{\nu}_0\delta(f) + \frac{a}{2}\big[\delta(f-f_m) + \delta(f+f_m)\big]$, the expression for the response of the network becomes

$$\begin{aligned} \mathcal{F}\bar{\lambda}(f) &= \frac{N_K \bar{K}\mathcal{F}\epsilon(0)\hat{\nu}_0\delta(f)}{1 - \tilde{N}_J \mathcal{F}\bar{\mathcal{J}}(0)\mathcal{F}\epsilon(0)} + \frac{a N_K \bar{K} e^{-2\pi i \hat{d} f}\mathcal{F}\epsilon(f)\big[\delta(f-f_m) + \delta(f+f_m)\big]}{2\big[1 - \tilde{N}_J \mathcal{F}\bar{\mathcal{J}}(f)\mathcal{F}\epsilon(f)\big]} \\ &= \frac{N_K \bar{K}\hat{\nu}_0\delta(f)}{1 - \tilde{N}_J \mathcal{F}\bar{\mathcal{J}}(0)} + \frac{a N_K \bar{K} e^{-2\pi i \hat{d} f_m}\mathcal{F}\epsilon(f_m)\delta(f-f_m)}{2\big[1 - \tilde{N}_J \mathcal{F}\bar{\mathcal{J}}(f_m)\mathcal{F}\epsilon(f_m)\big]} + \frac{a N_K \bar{K} e^{2\pi i \hat{d} f_m}\mathcal{F}\epsilon(-f_m)\delta(f+f_m)}{2\big[1 - \tilde{N}_J \mathcal{F}\bar{\mathcal{J}}(-f_m)\mathcal{F}\epsilon(-f_m)\big]} \\ \bar{\lambda}(t) &= \frac{N_K \bar{K}\hat{\nu}_0}{1 - \tilde{N}_J \mathcal{F}\bar{\mathcal{J}}(0)} + \frac{a N_K \bar{K} e^{-2\pi i \hat{d} f_m}\mathcal{F}\epsilon(f_m)e^{2\pi i f_m t}}{2\big[1 - \tilde{N}_J \mathcal{F}\bar{\mathcal{J}}(f_m)\mathcal{F}\epsilon(f_m)\big]} + \frac{a N_K \bar{K} e^{2\pi i \hat{d} f_m}\mathcal{F}\epsilon(-f_m)e^{-2\pi i f_m t}}{2\big[1 - \tilde{N}_J \mathcal{F}\bar{\mathcal{J}}(-f_m)\mathcal{F}\epsilon(-f_m)\big]} \\ &= \bar{\nu} + a N_K \bar{K}\,\mathrm{Re}\left[\frac{\mathcal{F}\epsilon(f_m)e^{2\pi i f_m (t+\hat{d})}}{1 - \tilde{N}_J \mathcal{F}\bar{\mathcal{J}}(f_m)\mathcal{F}\epsilon(f_m)}\right] \\ &= \bar{\nu} + a N_K \bar{K} r_\epsilon(f_m)\,\mathrm{Re}\left\{\frac{e^{i[2\pi f_m (t-\hat{d}) - \phi_\epsilon(f_m)]}}{1 - r_\epsilon(f_m)e^{-i\phi_\epsilon(f_m)}\tilde{N}_J r_{\bar{\mathcal{J}}}(f_m)e^{-i\phi_{\bar{\mathcal{J}}}(f_m)}}\right\}, \end{aligned} \tag{18}$$

where $\mathcal{F}\epsilon(f) = r_\epsilon(f)e^{-i\phi_\epsilon(f)}$, $\mathcal{F}\mathcal{J}(f) = \int_{d_{\min}}^{d_{\max}} \mathcal{J}(x)e^{-2\pi i f x}dx = r_{\bar{\mathcal{J}}}(f)e^{-i\phi_{\bar{\mathcal{J}}}(f)}$, and $\bar{\nu} = \frac{N_K \bar{K}\hat{\nu}_0}{1 - \tilde{N}_J \mathcal{F}\bar{\mathcal{J}}(0)} = \frac{N_K \bar{K}\hat{\nu}_0}{1 - N_J \bar{J}}$. This gives Equation (22) in the main text.

5 Network Response for Two Groups

For two recurrently connected groups where the within group weights have been depressed each of the group responses are given in Equation (38) of main text. The Fourier transforms of these is

$$\begin{aligned} \mathcal{F}\bar{\lambda}_1(f) &= \tilde{N}_J \mathcal{F}\bar{\mathcal{J}}_{12}(f)\mathcal{F}\epsilon(f)\mathcal{F}\bar{\lambda}_2(f) + N_K \bar{K} e^{-2\pi i \hat{d} f}\mathcal{F}\epsilon(f)\mathcal{F}\hat{\lambda}_1(f), \\ \mathcal{F}\bar{\lambda}_2(f) &= \tilde{N}_J \mathcal{F}\bar{\mathcal{J}}_{21}(f)\mathcal{F}\epsilon(f)\mathcal{F}\bar{\lambda}_1(f) + N_K \bar{K} e^{-2\pi i \hat{d} f}\mathcal{F}\epsilon(f)\mathcal{F}\hat{\lambda}_2(f), \end{aligned} \tag{19}$$

and by rearranging these we get

$$\begin{aligned} \mathcal{F}\bar{\lambda}_1(f) &= \frac{N_K \bar{K} e^{-2\pi i \hat{d} f}\mathcal{F}\epsilon(f)\Big[\mathcal{F}\epsilon(f)\tilde{N}_J \mathcal{F}\bar{\mathcal{J}}_{12}(f)\mathcal{F}\hat{\lambda}_2(f) + \mathcal{F}\hat{\lambda}_1(f)\Big]}{1 - \mathcal{F}\epsilon^2(f)\tilde{N}_J^2 \mathcal{F}\bar{\mathcal{J}}_{12}(f)\mathcal{F}\bar{\mathcal{J}}_{21}(f)}, \\ \mathcal{F}\bar{\lambda}_2(f) &= \frac{N_K \bar{K} e^{-2\pi i \hat{d} f}\mathcal{F}\epsilon(f)\Big[\mathcal{F}\epsilon(f)\tilde{N}_J \mathcal{F}\bar{\mathcal{J}}_{21}(f)\mathcal{F}\hat{\lambda}_1(f) + \mathcal{F}\hat{\lambda}_2(f)\Big]}{1 - \mathcal{F}\epsilon^2(f)\tilde{N}_J^2 \mathcal{F}\bar{\mathcal{J}}_{21}(f)\mathcal{F}\bar{\mathcal{J}}_{12}(f)}, \end{aligned} \tag{20}$$

which can be approximated as

$$
\begin{aligned}
\mathcal{F}\bar{\lambda}_1(f) \approx\ & aN_K\bar{K}e^{-2\pi i\hat{d}f}r_\epsilon(f)e^{-i\phi_\epsilon(f)}\Big[1 + r_\epsilon(f)e^{-i\phi_\epsilon(f)}\tilde{N}_J r_{\bar{\mathcal{J}}_{12}}(f)e^{-i\phi_{\bar{\mathcal{J}}_{12}}(f)}e^{-2\pi i\hat{d}_{\text{lag}}f} \\
& + r_\epsilon^2(f)e^{-2i\phi_\epsilon(f)}\tilde{N}_J r_{\bar{\mathcal{J}}_{12}}(f)e^{-i\phi_{\bar{\mathcal{J}}_{12}}(f)}\tilde{N}_J r_{\bar{\mathcal{J}}_{21}}(f)e^{-i\phi_{\bar{\mathcal{J}}_{21}}(f)}\Big]\Big[\delta(f - f_{\text{m}}) + \delta(f + f_{\text{m}})\Big], \\
\mathcal{F}\bar{\lambda}_2(f) \approx\ & aN_K\bar{K}e^{-2\pi i(\hat{d}+\hat{d}_{\text{lag}})f}r_\epsilon(f)e^{-i\phi_\epsilon(f)}\Big[1 + r_\epsilon(f)e^{-i\phi_\epsilon(f)}\tilde{N}_J r_{\bar{\mathcal{J}}_{21}}(f)e^{-i\phi_{\bar{\mathcal{J}}_{21}}(f)}e^{2\pi i\hat{d}_{\text{lag}}f} \\
& + r_\epsilon^2(f)e^{-2i\phi_\epsilon(f)}\tilde{N}_J r_{\bar{\mathcal{J}}_{21}}(f)e^{-i\phi_{\bar{\mathcal{J}}_{21}}(f)}\tilde{N}_J r_{\bar{\mathcal{J}}_{12}}(f)e^{-i\phi_{\bar{\mathcal{J}}_{12}}(f)}\Big]\Big[\delta(f - f_{\text{m}}) + \delta(f + f_{\text{m}})\Big].
\end{aligned}
\tag{21}
$$

This is then used to give Equation (39) in main text.

6 Learning Window and EPSP Kernel

It is assumed that $W(u)$ and $\epsilon(u)$ are given by

$$W(u) = -c_d e^{-\frac{u}{\tau_d}}h(u) + c_p e^{\frac{u}{\tau_p}}h(-u), \tag{22}$$

and

$$\epsilon(u) = \frac{1}{\tau_B - \tau_A}\left(e^{-\frac{u}{\tau_B}} - e^{-\frac{u}{\tau_A}}\right)h(u), \tag{23}$$

where $\tau_B > \tau_A$. From this, it can be seen that

$$
\begin{aligned}
\mathcal{F}W(f) &= \frac{c_p\tau_p}{1 - 2\pi i\tau_p f} - \frac{c_d\tau_d}{1 + 2\pi i\tau_d f} \\
&= \frac{c_p\tau_p}{1 + 4\pi^2\tau_p^2 f^2} - \frac{c_d\tau_d}{1 + 4\pi^2\tau_d^2 f^2} + 2\pi i f\left(\frac{c_p\tau_p^2}{1 + 4\pi^2\tau_p^2 f^2} + \frac{c_d\tau_d^2}{1 + 4\pi^2\tau_d^2 f^2}\right) \\
&= \frac{c_p\tau_p - c_d\tau_d + 4\pi^2 f^2\tau_p\tau_d(c_p\tau_d - c_d\tau_p) + 2\pi i f\left[c_p\tau_p^2 + c_d\tau_d^2 + 4\pi^2\omega^2\tau_p^2\tau_d^2(c_p + c_d)\right]}{(1 + 4\pi^2\tau_p^2 f^2)(1 + 4\pi^2\tau_d^2 f^2)},
\end{aligned}
\tag{24}
$$

and

$$
\begin{aligned}
\mathcal{F}\epsilon(f) &= \frac{1}{\tau_B - \tau_A}\left(\frac{\tau_B}{1 + 2\pi i\tau_B f} - \frac{\tau_A}{1 + 2\pi i\tau_A f}\right) \\
&= \frac{1}{(\tau_B - \tau_A)}\left[\frac{\tau_B}{1 + 4\pi^2\tau_B^2 f^2} - \frac{\tau_A}{1 + 4\pi^2\tau_A^2 f^2} - 2\pi i f\left(\frac{\tau_B^2}{1 + 4\pi^2\tau_B^2 f^2} - \frac{\tau_A^2}{1 + 4\pi^2\tau_A^2 f^2}\right)\right] \\
&= \frac{(\tau_B - \tau_A) + 4\pi^2 f^2\tau_B\tau_A(\tau_B^2 - \tau_A^2) - 2\pi i f(\tau_B^2 - \tau_A^2)}{(\tau_B - \tau_A)(1 + 4\pi^2\tau_B^2 f^2)(1 + 4\pi^2\tau_A^2 f^2)}.
\end{aligned}
\tag{25}
$$

It can be seen that $\mathcal{F}W(-f) = (\mathcal{F}W(f))^*$ and $\mathcal{F}\epsilon(-f) = (\mathcal{F}\epsilon(f))^*$. Writing $\mathcal{F}W(f)$ in polar form gives

$$\mathcal{F}W(f) = r_W(f)e^{i\phi_W(f)}, \tag{26}$$

where

$$
\begin{aligned}
r_W(f) &= \frac{\sqrt{\left[c_p\tau_p - c_d\tau_d + 4\pi^2 f^2\tau_p\tau_d(c_p\tau_d - c_d\tau_p)\right]^2 + 4\pi^2 f^2\left[c_p\tau_p^2 + c_d\tau_d^2 + 4\pi^2 f^2\tau_p^2\tau_d^2(c_p + c_d)\right]^2}}{(1 + 4\pi^2\tau_p^2 f^2)(1 + 4\pi^2\tau_d^2 f^2)}, \\
\phi_W(f) &= \begin{cases} \arctan(\frac{x}{y}) & \text{for } y > 0 \\ \frac{\pi}{2} & \text{for } y = 0 \\ \arctan(\frac{x}{y}) + \pi & \text{for } y < 0 \end{cases},
\end{aligned}
\tag{27}
$$

where $x = 2\pi f[c_p\tau_p^2 + c_d\tau_d^2 + 4\pi^2 f^2\tau_p^2\tau_d^2(c_p + c_d)]$ and $y = c_p\tau_p - c_d\tau_d + 4\pi^2 f^2\tau_p\tau_d(c_p\tau_d - c_d\tau_p)$. Plots of $r_W(f)$ and $\phi_W(f)$ are shown in Figures S1 and 5B, respectively. Writing $\mathcal{F}\epsilon(f)$ in polar form gives

$$\mathcal{F}\epsilon(f) = r_\epsilon(f)e^{-i\phi_\epsilon(f)}, \tag{28}$$

where

$$
\begin{aligned}
r_\epsilon(f) &= \frac{\sqrt{\left[(\tau_B - \tau_A) + 4\pi^2 f^2 \tau_B \tau_A (\tau_B^2 - \tau_A^2)\right]^2 + 4\pi^2 f^2 (\tau_B^2 - \tau_A^2)^2}}{(\tau_B - \tau_A)(1 + 4\pi^2 \tau_B^2 f^2)(1 + 4\pi^2 \tau_A^2 f^2)} \\
&= \frac{\sqrt{1 + 8\pi^2 f^2 \tau_B \tau_A (\tau_B + \tau_A) + 16\pi^4 f^4 \tau_B^2 \tau_A^2 (\tau_B + \tau_A)^2 + 4\pi^2 f^2 (\tau_B + \tau_A)^2}}{(1 + 4\pi^2 \tau_B^2 f^2)(1 + 4\pi^2 \tau_A^2 f^2)} \\
&= \frac{\sqrt{1 + 4\pi^2 f^2 (\tau_B + \tau_A)\left[2\tau_B \tau_A + 4\pi^2 f^2 \tau_B^2 \tau_A^2 (\tau_B + \tau_A) + \tau_B + \tau_A\right]}}{(1 + 4\pi^2 \tau_B^2 f^2)(1 + 4\pi^2 \tau_A^2 f^2)}, \\
\phi_\epsilon(f) &= \arctan\left[\frac{2\pi f(\tau_B^2 - \tau_A^2)}{(\tau_B - \tau_A) + 4\pi^2 f^2 \tau_B \tau_A (\tau_B^2 - \tau_A^2)}\right] \\
&= \arctan\left[\frac{2\pi f(\tau_B + \tau_A)}{1 + 4\pi^2 f^2 \tau_B \tau_A (\tau_B + \tau_A)}\right].
\end{aligned}
\tag{29}
$$

It can be seen from this that $\mathcal{F}W(0) = \tilde{W}$ and $\mathcal{F}\epsilon(0) = 1$. Plots of $r_\epsilon(f)$ and $\phi_\epsilon(f)$ are shown in Figure 5A and B, respectively.

7 Estimating the Amplitude of a Sum of Cosines

The amplitude of

$$
S(x) = \cos(x + a) + \sum_i B_i \cos(x + b_i), \tag{30}
$$

is unchanged under a shift in the x axis. So

$$
S(x - a) = \cos(x) + \sum_i B_i \cos(x + b'_i), \tag{31}
$$

where $b'_i = b_i - a$, will have the same amplitude. This can be written as

$$
\begin{aligned}
S(x - a) &= \cos(x) + \sum_i \left[B_i \cos(b'_i)\cos(x) - B_i \sin(b'_i)\sin(x)\right] \\
&= \left[1 + \sum_i B_i \cos(b'_i)\right]\cos(x) - \left[\sum_i B_i \sin(b'_i)\right]\sin(x) \\
&= P\cos(x) + Q\sin(x),
\end{aligned}
\tag{32}
$$

where $P = 1 + \sum_i B_i \cos(b'_i)$ and $Q = -\sum_i B_i \sin(b'_i)$. This can be written in the form

$$
S(x - a) = W\cos(x + \gamma), \tag{33}
$$

where the amplitude, W, is given by

$$
\begin{aligned}
W^2 = P^2 + Q^2 &= \left[1 + \sum_i B_i \cos(b'_i)\right]^2 + \left[\sum_i B_i \sin(b'_i)\right]^2 \\
&= 1 + 2\sum_i B_i \cos(b'_i) + 2\sum_{i,j\neq i} B_i B_j \cos(b'_i)\cos(b'_j) + \sum_i B_i^2 \cos^2(b'_i) \\
&\quad + \sum_i B_i^2 \sin^2(b'_i) + 2\sum_{i,j\neq i} B_i B_j \sin(b'_i)\sin(b'_j) \\
&= 1 + 2\sum_i B_i \cos(b'_i) + \sum_i B_i^2 + \sum_{i,j\neq i} B_i B_j \left[\cos(b'_i - b'_j) + \cos(b'_i + b'_j)\right] \\
&\quad + \sum_{i,j\neq i} B_i B_j \left[\cos(b'_i - b'_j) - \cos(b'_i + b'_j)\right] \\
&= 1 + 2\sum_i B_i \cos(b'_i) + \sum_i B_i^2 + 2\sum_{i,j\neq i} B_i B_j \cos(b'_i - b'_j) \\
&= 1 + \sum_i B_i \left[2\cos(b_i - a) + B_i + 2\sum_{j\neq i} B_j \cos(b_i - b_j)\right],
\end{aligned}
\tag{34}
$$

and so

$$W = \sqrt{1 + \sum_i B_i \left[2\cos(b_i - a) + B_i + 2\sum_{j \neq i} B_j \cos(b_i - b_j) \right]}. \tag{35}$$

For the case where we have $B_i \propto X^i$, $X < 1$, and it is an infinite sum of cosines, we can estimate the square of the amplitude to the $(k+1)$th order with

$$W^2 = 1 + 2\sum_i^k B_i \cos(b_i - a) + \sum_i^{\lfloor k/2 \rfloor} B_i^2 + 2\sum_i^k \sum_{j \neq i}^{k-i} B_i B_j \cos(b_i - b_j), \tag{36}$$

where $\lfloor x \rfloor$ is the floor of x.

8 Third-Order Covariance of Oscillatory Inputs

Similar to the second-order input covariance,

$$\hat{C}_{kl}(t,u) = \frac{1}{T}\int_{t-T}^{t} \langle \hat{S}_k(t')\hat{S}_l(t'+u)\rangle dt' - \left(\frac{1}{T}\int_{t-T}^{t} \langle \hat{S}_k(t')\rangle dt'\right)\left(\frac{1}{T}\int_{t-T}^{t} \langle \hat{S}_l(t'+u)\rangle dt'\right), \tag{37}$$

we defined the third-order input covariance as

$$\begin{aligned}
\hat{C}_{klm}(t,u,r) = \frac{1}{T}\int_{t-T}^{t} & \langle \hat{S}_k(t')\hat{S}_l(t'+u)\hat{S}_m(t'+u+r)\rangle dt' \\
& - \left(\frac{1}{T}\int_{t-T}^{t} \langle \hat{S}_k(t')\rangle dt'\right)\left(\frac{1}{T}\int_{t-T}^{t} \langle \hat{S}_l(t'+u)\hat{S}_m(t'+u+r)\rangle dt'\right) \\
& - \left(\frac{1}{T}\int_{t-T}^{t} \langle \hat{S}_l(t'+u)\rangle dt'\right)\left(\frac{1}{T}\int_{t-T}^{t} \langle \hat{S}_k(t')\hat{S}_m(t'+u+r)\rangle dt'\right) \\
& - \left(\frac{1}{T}\int_{t-T}^{t} \langle \hat{S}_m(t'+u+r)\rangle dt'\right)\left(\frac{1}{T}\int_{t-T}^{t} \langle \hat{S}_k(t')\hat{S}_l(t'+u)\rangle dt'\right) \\
& - \left(\frac{1}{T}\int_{t-T}^{t} \langle \hat{S}_k(t')\rangle dt'\right)\left(\frac{1}{T}\int_{t-T}^{t} \langle \hat{S}_l(t'+u)\rangle dt'\right)\left(\frac{1}{T}\int_{t-T}^{t} \langle \hat{S}_m(t'+u+r)\rangle dt'\right) \\
= \frac{1}{T}\int_{t-T}^{t} & \langle \hat{S}_k(t')\hat{S}_l(t'+u)\hat{S}_m(t'+u+r)\rangle dt' \\
& - \hat{\nu}_k \hat{C}_{lm}(t+u,r) - \hat{\nu}_l \hat{C}_{km}(t,u+r) - \hat{\nu}_m \hat{C}_{kl}(t,u) - \hat{\nu}_k\hat{\nu}_l\hat{\nu}_m.
\end{aligned} \tag{38}$$

So for inputs which are simple realizations of identical, sinusoidal intensity functions given by $\hat{\nu}_0 + a\cos(2\pi f_m t)$, this is

$$\begin{aligned}
\hat{C}_{klm}(t,u,r) &= \frac{1}{T}\int_{t-T}^{t} \{\hat{\nu}_0 + a\cos[2\pi f_m t']\}\{\hat{\nu}_0 + a\cos[2\pi f_m(t'+u)]\}\{\hat{\nu}_0 + a\cos[2\pi f_m(t'+u+r)]\} dt' \\
&\quad - \frac{a^2\hat{\nu}_0}{2}\Big\{\cos[2\pi f_m r] + \cos[2\pi f_m(u+r)] + \cos[2\pi f_m u]\Big\} - \hat{\nu}_0^3 \\
&= \frac{a^3}{T}\int_{t-T}^{t} \cos[2\pi f_m t']\cos[2\pi f_m(t'+u)]\cos[2\pi f_m(t'+u+r)] dt' \\
&= \frac{a^3}{2T}\int_{t-T}^{t} \Big\{\cos[2\pi f_m u] + \cos[2\pi f_m(2t'+u)]\Big\}\cos[2\pi f_m(t'+u+r)] dt' \\
&= \frac{a^3}{4T}\int_{t-T}^{t} \Big\{\cos[2\pi f_m(t'+r)] + \cos[2\pi f_m(t'+2u+r)] + \cos[2\pi f_m(t'-r)] \\
&\quad + \cos[2\pi f_m(3t'+2u+r)]\Big\} dt' \\
&= 0.
\end{aligned} \tag{39}$$

References

1. Gilson M, Burkitt AN, Grayden DB, Thomas DA, van Hemmen JL (2009) Emergence of network structure due to spike-timing-dependent plasticity in recurrent neuronal networks IV: Structuring synaptic pathways among recurrent connections. Biol Cybern 101: 427–444.
